# Dorsal raphe nucleus controls motivation-state transitions in monkeys

**DOI:** 10.1126/sciadv.ads1236

**Published:** 2025-06-27

**Authors:** Luke Priestley, Mark Chiew, Mo Shahdloo, Ali Mahmoodi, Xinghao Cheng, Robin Cleveland, Matthew Rushworth, Nima Khalighinejad

**Affiliations:** ^1^ Wellcome Centre for Integrative Neuroimaging, Department of Experimental Psychology, University of Oxford, Tinsley Building, Oxford, UK.; ^2^ Wellcome Centre for Integrative Neuroimaging, Oxford Centre for Functional MRI of the Brain (FMRIB), Nuffield Department of Clinical Neurosciences, University of Oxford, John Radcliffe Hospital, Oxford, UK.; ^3^ Oxford institute of Biomedical Engineering, Department of Engineering Science, University of Oxford, Old Road Campus Research Building, Oxford, UK.

## Abstract

The dorsal raphe nucleus (DRN) is an important source of serotonin in the brain, but fundamental aspects of its function remain elusive. Here, we present a combination of minimally invasive recording and disruption studies to show that DRN brings about changes in motivation states. We use recently developed methods for identifying temporal patterns in behavior to show that monkeys change their motivation depending on the availability of rewards in the environment. Distinctive patterns of DRN activity occur when monkeys transition between a high-motivation state occupied when rewards are abundant, to a low-motivation state engendered by reward scarcity. Disrupting DRN diminishes sensitivity to the reward environment and perturbs transitions in motivational states.

## Teaser

The brain’s key serotonergic nucleus controls changes in motivation that reconcile behavior with the surrounding world.

## INTRODUCTION

Animals need rewards for their survival, and they need to obtain them as efficiently as possible. In many naturalistic scenarios, this means tracking general features of the surrounding environment: The foraging behavior of many species, for example, involves comparing the opportunity an animal is currently confronted with against the general richness and stochasticity of opportunities it has encountered in the recent past, which guide its expectations for the future (*1*, *2*). These are rational considerations for animals given the biological constraints that encumber them. Finding and pursuing rewards consumes precious metabolic resources that must later be replenished, and so it is critical that animals organize their reward-seeking activities in ways that exploit their external milieu—to press their advantage when things are good and conserve their energy when things are bad.

How does the brain reconcile motivation for rewards with the environment in this way? Here, we argue for a critical role for the dorsal raphe nucleus (DRN)—a phylogenetically ancient part of the brainstem that is distinguished by its serotonergic innervation of the mammalian forebrain. Although fundamental aspects of DRN’s function remain elusive, two major themes are discernible: (i) that DRN controls changes in an animal’s behavior and (ii) that DRN responds to reward-related features of an animal’s milieu, like the value, valence, and uncertainty of recent outcomes (*3*–*10*).

We build on this previous work in arguing that DRN controls transitions between motivational states that reconcile an animal’s behavior with the distribution of rewards in the environment. We use a novel behavioral paradigm to demonstrate that rhesus monkeys are more motivated to pursue rewards that occur in rich environments with many high-value opportunities. We implement recent innovations in quantitative behavioral modeling to show that this pattern is explicable by state-like changes in an animal’s motivation that match the current environment’s reward distribution. We take advantage of the whole-brain perspective afforded by functional magnetic resonance imaging (fMRI) to show that brain activity in DRN—but no other neuromodulatory nucleus—covaries with transitions in motivation-states, specifically when monkeys transition between a high-motivation state occupied when rewards are abundant, to a low-motivation state engendered by reward scarcity. Such comparisons are critical for identifying the different functions of different neuromodulatory nuclei (*11*). Interpreting the causal significance of fMRI activation patterns is possible when the impact of disrupting the activity can be examined (*12*–*14*). Accordingly, we modulate neural activity with minimally invasive transcranial ultrasound stimulation (TUS) to show that DRN, but not a functionally related neuromodulatory nucleus—ventral tegmental area (VTA)—is causally involved in motivational-state transitions (*15*–*17*). In doing so, we provide the first demonstration that minimally invasive modulation of DRN is possible and a new perspective on DRN’s behavioral function.

## RESULTS

### Animal behavior is modulated by the environment

**Fig. 1.**
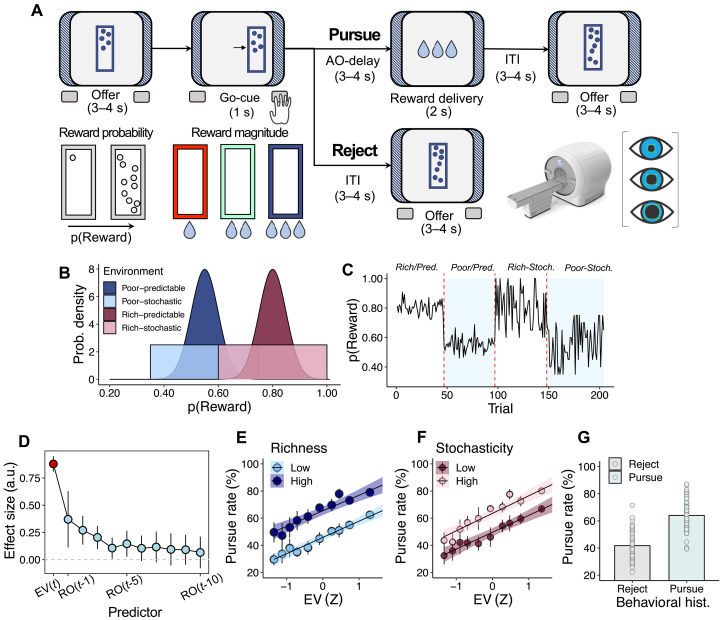
Animals are more likely to pursue reward opportunities in rich environments. (**A**) The behavioral task involved sequential encounters with reward opportunities that varied in reward magnitude (stimulus color) and reward probability (dots per stimulus). Animals performed the task while undergoing functional MRI and pupillometry. (**B**) The probability density of reward probability in different reward environments. Rich blocks [purple; mean(reward probability) = 0.80] had higher mean reward probability than poor blocks [blue; mean(reward probability) = 0.55]. Stochastic blocks [light colors; SD(reward probability) = 0.13] were uniformly distributed, and predictable blocks were normally distributed [dark colors; SD(reward-probability) = 0.05]. Block-type was cued with a visual stimulus bordering the screen (see Materials and Methods). (**C**) A depiction of changes in reward probability as a function of reward environment in an example session. Each session comprised four environments covering each permutation of richness and stochasticity. (**D**) Effect size of reward outcomes (RO) on past trials on pursue-versus-reject decision on the current trial. For comparison, the effect size of the expected value (EV) of the current trial is shown with a red point. Points and whiskers indicate effect size (i.e., regression weight) ± 95% confidence interval (CI) for each predictor. Effects for outcomes past *t* – 5 are nonsignificant at 0.05 level. (**E**) Animals were more likely to pursue opportunities as the richness of the environment increased. *x* axis indicates expected value of the reward opportunity on each trial. *y* axis indicates rate of reward pursuit. Color scale indicates mean-split according to richness of the environment, i.e., high is trials where richness_*t*_ > μ_richness_ and low is richness_*t*_ ≤ μ_richness_. Dots and whiskers indicate means (±SEM) of the pursue rate in deciles expected value over each animal in each session. (**F**) Animals were more likely to pursue opportunities as the stochasticity of the environment increased. *x* axis, *y* axis, dots, whiskers, and color scale follow conventions of (E). (**G**) Animals were likely to repeat pursue-versus-reject decisions over consecutive trials. Dots indicate mean level of responding per animal per session. a.u., arbitrary units.

Four rhesus monkeys (*Macaca mulatta*) performed a simple decision-making task involving sequential encounters with reward opportunities that varied in reward magnitude and reward probability (Fig. 1A). Upon each encounter, the animals could either pursue the opportunity and incur a short temporal cost or let the opportunity pass and proceed to the next encounter. Each session comprised four blocks of 40 to 50 trials. The mean reaction time (RT) after the go cue was 0.76 s (SD = 0.07 s). The mean premature response rate was 3% (SD = 1.8%) of trials per session. The animals performed one session per day at the same time of the day.

We systematically controlled the reward probability values for reward opportunities within each block to engender different reward environments. In other words, the distribution of a given reward-probability value varied between blocks. Blocks varied on two dimensions: (i) richness, defined by the mean reward probability of opportunities—i.e., the value of an average opportunity in a block, and (ii) stochasticity, defined as trial-to-trial variability in reward probability, and implemented by changing the width of reward-probability distributions (Fig. 1, B and C; see Materials and Methods for details). We refer to the reward probability associated with a specific reward opportunity as reward probability. We refer to blocks in which reward probabilities were higher, on average, as rich blocks, and to blocks in which they were lower on average as poor blocks. Similarly, we refer to blocks in which there was high variance in reward-probability values as stochastic blocks, and to blocks in which there was low variance as predictable blocks.

We first confirmed that animals were more likely to pursue reward opportunities as a function of increases in an opportunity’s reward probability (fig. S1, A-i) and reward magnitude (fig. S1, A-ii). Then, we examined how animals changed their behavior in response to the environment. We reasoned that an animal’s understanding of the environment would depend on its reward history. To determine the time horizon of sensitivity to past rewards, we used a mixed-effects logistic regression with individual terms for reward outcomes on trials *t –* 1 to *t –* 10 (where *t* is the current trial; GLM1.1, see Materials and Methods). This suggested that behavior was modulated by rewards as distant as five trials into the past, but no further (Fig. 1D). We therefore operationalized the richness of the environment as the average reward accumulated in the previous five trials and the stochasticity of the environment as the SD of the reward rate over the same five trial period. Changing the time window used to operationalize environments, or operationalizing the environment using the blocks specified in the experimental design rather than reward history, did not affect the inferences drawn from any following analyses (see figs. S1, B and C, and S2).

We quantified the influence that the richness and stochasticity of the environment had on behavior with a series of mixed-effect binomial general linear models (GLM) that accounted for animal-specific variation in behavior. This revealed that animals were more likely to pursue opportunities as a function of increases in the richness and stochasticity of the environment (GLM1.2, see Materials and Methods; β_environment-richness_ = 0.46, SE = 0.08, *P* < 0.001; Fig. 1E; see also fig. S1, B to D; β_environment-stochasticity_ = 0.08, SE = 0.03, *P* = 0.020; Fig. 1F; see also fig. S1E). These effects were independent of trial-by-trial reward value—For example, opportunities with the same expectation value were more likely to be pursued in rich relative to poor environments, meaning that the effect was not due to the correlation between rich environments and high-value reward opportunities on a given trial (Fig. 1, E and F; see also fig. S1, B, D, and E).

The fact that the richness of the environment predicted whether animals would pursue reward opportunities indicated a subtle but important feature of behavior. Animals could only obtain rewards by pursuing reward opportunities, meaning that rich environments effectively corresponded to periods where many rewards had been pursued in the recent past. Insofar as receiving rewards in the past encouraged animals to pursue opportunities in the present, the richness of the environment effect therefore indicated that pursue-versus-reject decisions were autocorrelated over consecutive trials. We tested this hypothesis by predicting the pursue-versus-reject decision taken on each trial using the corresponding decision on the previous trial (behavioral history, hence). This confirmed that animals tended to repeat pursue-versus-reject decisions over consecutive trials, although each trial featured a separate reward opportunity with distinct magnitude and probability parameters (GLM1.3; β_behavioral-history_ = 0.64, SE = 0.24, *P* < 0.001; Fig. 1G).

### A hidden Markov model identifies motivation states in behavior

**Fig. 2.**
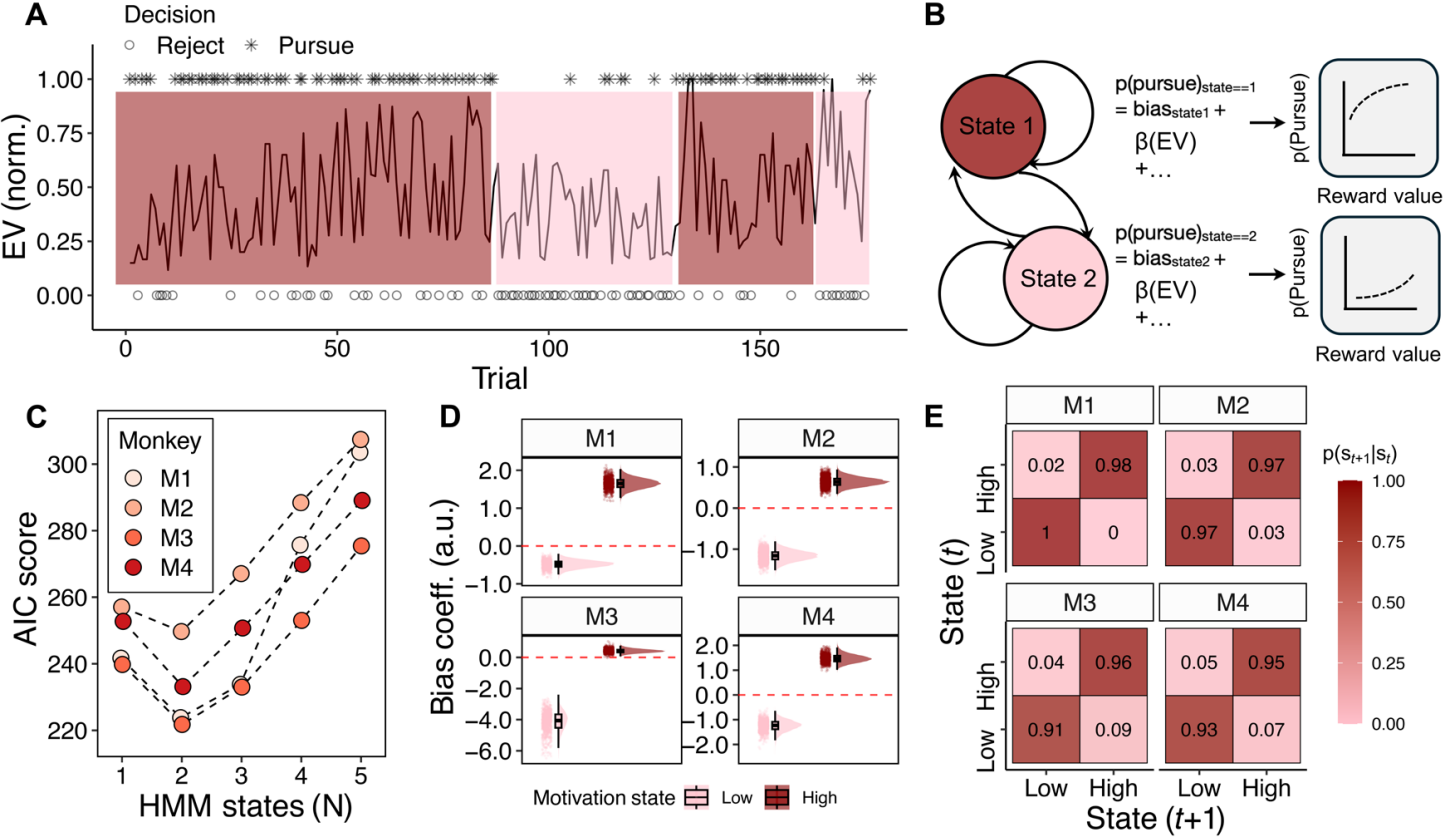
A general linear model–hidden Markov model for animal behavior. (**A**) Pursue-versus-reject decisions (stars at the top indicate reward pursuits; dots at the bottom indicate reward rejections) as a function of expectation value (*y* axis) over time (*x* axis) in an example session. The animals showed prolonged biases to pursue (red) or reject (pink) opportunities over many consecutive trials. (**B**) We captured these patterns using a GLM-HMM that featured state-dependent bias parameters. These bias parameters produced state-dependent decision functions that reflected changes in the way animals made pursue-versus-reject decisions over time. (**C**) A two-state GLM–HMM improved fit relative to other models according to AIC—a penalized form of log-likelihood (see fig. S3 for full cross-validation results). Points show mean session-wise AIC for each animal. (**D**) The posterior probabilities of state-specific bias parameter values in each animal (M1–M4) suggested that HMM-states were associated with meaningful changes in an animal’s bias to pursue-versus-reject rewards. (**E**) Transition matrices indicated that in each animal, HMM-states were strongly autocorrelated over consecutive trials (M1–M4).

We reasoned that autocorrelations in animal behavior might reflect changes in motivation states—that is, changes in an animal’s intrinsic propensity to pursue rewards that are independent of the value of the opportunity currently at stake and that might persist over many trials (Fig. 2, A and B). It has recently been shown that latent time-varying dispositions in behavior can be uncovered using a general linear model hidden Markov model (GLM-HMM) approach (*18*, *19*). We therefore tested the motivation-state hypothesis using a GLM-HMM.

GLM-HMMs are an extension of the classical GLM in which the parameter values of a GLM are allowed to change over time-varying latent states. Transitions between latent states are governed by Markovian dynamics and are thus called “HMM states.” In simple terms, this means that if animals behave in fundamentally different ways—for example, if they adopt different motivation states—over the course of the task, the GLM-HMM can detect these states and specify how the relationship between the task and behavior changes in each state via the weights in the GLM part of the model. We formulated a GLM-HMM where the pursue-reject decision on each trial was predicted with a binomial GLM that included the following terms: (i) a bias or “intercept” term, (ii) a predictor for the expectation value of the reward opportunity available on each trial, and (iii) predictors for external environment cues that were presented throughout the task (Fig. 2B and fig. S3; see Materials and Methods for details). Our hypothesis was that animals’ experienced changes in their intrinsic predisposition to pursue-versus-reject reward opportunities, over and above any influence of extrinsic factors such as the expectation value of the current opportunity. We therefore let only the bias parameter change between HMM states and held the weights on other predictors constant (but see figs. S5 and S6 for alternative models in which additional parameters changed between HMM states). Incorporating predictors for task features in the GLM part of the model ensured that the behavioral changes identified by the GLM-HMM are specifically due to shifts in the animal’s intrinsic propensity to pursue rewards, after taking into account external factors like the reward value on the current trial.

We used a previously reported model-selection procedure, which involved testing models with different numbers of HMM states using fivefold cross-validation (see fig. S3 for details) (*18*). We validated the procedure using a series of parameter recovery tests (see fig. S8). After confirming successful parameter recovery, we implemented it on behavioral data. This indicated that cross-validated log-likelihoods for two-state GLM-HMMs were higher than one-state GLM-HMMs for all animals (Fig. 2C; see fig. S3 for full cross-validation results) but that invoking additional states beyond this number was not warranted for any animal. We therefore used two-state GLM-HMMs for all further analyses.

**Fig. 3.**
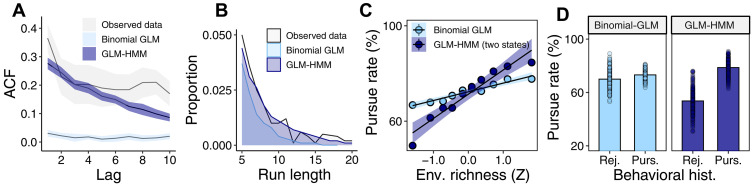
A two-state GLM-HMM recapitulates qualitative patterns of animal behavior. (**A**) Data simulated from a two-state GLM-HMM reproduced the autocorrelations in decision-making that characterized animal behavior. A binomial GLM did not reproduce this pattern. *y* axis shows autocorrelation function (ACF) between the decision on trial *t* and decisions on (*t* – 1):(*t* – 10) for recorded behavior, the two-state GLM-HMM and a binomial GLM for an example animal. Lines and shaded areas show the means and SEM, respectively, of the ACF at each individual lag. (**B**) A complementary perspective on autocorrelated decisions is decision run lengths—the number of times that a decision is repeated over consecutive trials [e.g., rejecting three consecutive opportunities is a run length of 3; see also (*18*)]. The GLM-HMM recapitulates observed patterns of run lengths, especially for run lengths spanning five or more consecutive trials. A binomial GLM is unable to reproduce this pattern. Data from the same example animal and simulations as previous panels. (**C** and **D**) Implementing the GLMs (GLM1.1 to 1.3) used to analyze behavior on simulated data from a two-state GLM-HMM recapitulated both richness of the environment (C) and behavioral history effects (D). The same effects did not occur in data simulated from one-binomial GLMs. See Fig. 1G and fig. S1D for comparison. In (C), dots indicate mean pursue rate in deciles of richness of the environment across simulations, and shaded area indicates SEM around line of best fit. In (D), dots indicate mean pursue rates in individual simulations.

Each animal-specific two-state GLM-HMM featured clearly distinct state-specific bias parameters (Fig. 2D) and profoundly autocorrelated HMM-state transition matrices, which were consistent with state-like fluctuations in motivation to pursue rewards (Fig. 2E). To further examine the validity of the model, we simulated data from a fitted two-state GLM-HMM and compared the output with: (i) the observed behavioral data recorded from animals and (ii) data simulated from a conventional binomial GLM with no HMM states. The two-state GLM-HMM data reproduced key features of animal behavior including trial-to-trial autocorrelation in pursue-versus-reject decisions, (Fig. 3, A and B) and the richness of the environment and behavioral history effects described earlier (Fig. 3, C and D). The binomial GLM with no HMM states was unable to reproduce this pattern. This demonstrates that the key behavioral effects described above—the tendency of animals to pursue more rewards in rich environments and to repeat their pursue-versus-reject decisions over time—can be explained parsimoniously by a two-state GLM-HMM in which there are time-varying changes in the bias term, which determines an animal’s predisposition to pursue or reject opportunities. Further analyses demonstrated that HMM states were not reducible to satiety, fatigue, or time on task (fig. S4).

**Fig. 4.**
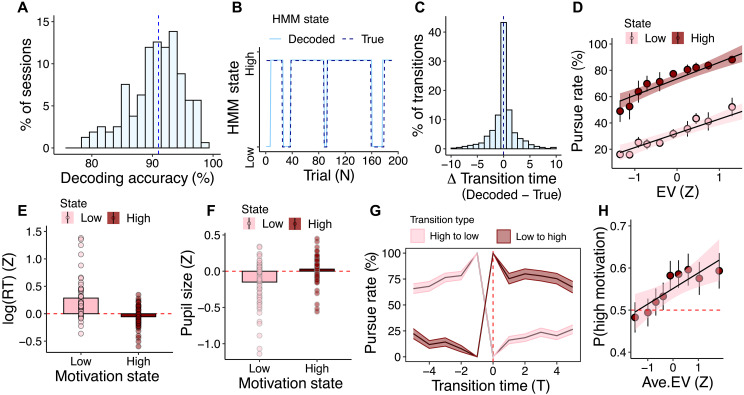
HMM states correspond to distinct, persistent, and biologically meaningful motivation states during behavior. We decoded the maximum a posteriori HMM state on each trial using the Viterbi algorithm and compared it to behavior. (**A**) We established Viterbi algorithm’s accuracy using simulated datasets where the true HMM state was known. Decoding was successful on 90.68% (±0.72%) of trials. Histogram shows distribution of session-level decoding accuracy (%) across simulated datasets. (**B**) Comparison of true and Viterbi-decoded states in example simulated session. (**C**) We assessed the accuracy of state-transition decoding by quantifying the distance between decoded and true transition trials. Most transition trials were exactly identified (mean distance = −0.03) and 84% of decoded transitions occurred within ±3 trial window of a true transition. Histogram shows proportion of decoded-versus-true transition distances. (**D**) Animals were more likely to pursue reward opportunities in the high-motivation state compared to the low-motivation state, over and above any influence of an opportunity’s expected value. Dots and whiskers indicate means ± SEM pursue rates in deciles of expected value. (**E**) RTs were faster when animals occupied the high-motivation state. Dots indicate mean RT for each animal in each session. (**F**) Pupil size during the decision phase (see Materials and Methods) of the task was greater when animals occupied the high-motivation state relative to the low-motivation state. Dots indicate mean pupil size in each animal in each session. (**G**) Pursue rates changed abruptly at motivation-state transitions. This suggests that the GLM-HMM identified state-like rather than gradual changes in behavior. Color shades indicate transition direction. Lines and shaded areas indicate means (±SEM) of pursue rate across all animals in all sessions (**H**) Animals are more likely to occupy the high-motivation state increases as the expectation value of recent reward opportunities (Ave. EV) increases. Dots and whiskers indicate means (±SEM) of rate of motivation-state occupancy in deciles average expectation value.

We tested the whether the statistical patterns identified by the GLM-HMM corresponded to biological motivation states with a series of follow-up analyses. To do this, we first decoded the maximum a posteriori HMM state on each trial using a well-established procedure called the Viterbi algorithm. We validated the Viterbi algorithm on simulated data, which demonstrated that it identified the correct HMM state on 90% of trials and identified HMM-state transitions within ± three trials of a true transition in 85% of cases (Fig. 4, A to C). We then examined how decoded HMM states were related to behavior. As expected, the animals were markedly more likely to pursue reward opportunities in the putative high-motivation state compared to the low-motivation state, over and above any influence of the reward value available (GLM2.1; β_motivation-level_ = 2.66, SE = 0.56, *P* < 0.001; Fig. 4D). Behavior during transitions between HMM states was characterized by abrupt changes in pursue rates, suggesting that the GLM-HMM captured state-like differences in behavior with a high degree of temporal precision (GLM2.2; β_before-versus-after-transition(high-to-low)_ = −1.48, SE = 0.08, *P* < 0.001; β_before-versus-after-transition(low-to-high)_ = 1.80, SE = 0.30, *P* < 0.001; Fig. 4G). Last, we established the model’s convergent validity by comparing HMM states to: (i) trial-by-trial pupil size, a well-validated indicator of physiological arousal (*20*), and (ii) trial-by-trial RTs for pursue decisions, a simple way of quantifying vigor. Consistent with the link between HMM states and motivation, putative high-motivation states featured faster RTs for pursue decisions (GLM2.3; β_motivation-level_ = −0.20, SE = 0.03, *P* < 0.001; Fig. 4E) and increases in pupil diameter during decision-making (GLM2.4; β_motivation-level_ = 0.20, SE = 0.02, *P* < 0.001; Fig. 4F). The GLM-HMM, thus, provided quantitative evidence for discrete, persistent, and biologically meaningful internal motivation-states in monkey behavior.

Last, we tested the hypothesis that motivation states were related to the external environment: Was it the case, for example, that animals matched their motivation state with the overarching distribution of rewards in the task milieu? We quantified the availability of rewards by calculating, for each trial, the average expectation value of reward opportunities in the preceding five trials. We did not use the richness of the environment metric introduced earlier because this was calculated on the basis of an animal’s reward history and was therefore contingent on its recent behavior—Relating an animal’s motivation state to its recent behavior, thus, would be a logically circular analysis. In contrast, the expectation value of reward opportunities was experimentally controlled via the reward distributions characterizing the task (Fig. 1B) and was therefore not affected by an animal’s decisions. This showed that animals were more likely to occupy high-motivation states as the expected value of recent opportunities increased (GLM2.5; β_Ave.EV_ = 0.19, SE = 0.08, *P* = 0.022; Fig. 4H; see also fig. S7). The GLM-HMM therefore demonstrated not just that animals experienced state-like changes in motivation for rewards but that these states were reconciled with the distribution of rewards in the world around them.

### Brain activity in DRN reflects an animal’s reward environment and changes in its motivation state

Animals performed the task under fMRI. Our analysis of fMRI recordings focused on a priori regions of interest (ROIs; see figs. S9 and S10) comprising the ascending neuromodulatory systems (ANS)—an assemblage of phylogenetically ancient nuclei that includes the serotonergic DRN, in addition to the VTA and substantia nigra (SN), the cholinergic nucleus basalis (NB), and the noradrenergic locus coeruleus (LC). We also examined habenula (Hb)—an epithalamic nucleus with diverse subcortical connections that interact with the ANS in a reciprocal fashion (*21*, *22*). Broadening our analysis beyond DRN provided a comparative perspective on its function, which is valuable given that reward functions are sometimes jointly attributed to different subcortical nuclei—The richness of the environment, for example, has been linked to both VTA and DRN-related signals (*9*, *10*, *23*, *24*), while various forms of reward uncertainty have been ascribed to DRN, LC, and the cholinergic basal forebrain alike (*11*, *25*–*28*). fMRI’s wholistic perspective enabled us to address this by comparing signals in DRN and other ANS nuclei (*12*). This was accomplished with a novel suite of fMRI acquisition and preprocessing methods that optimized blood oxygen level–dependent (BOLD) signal from subcortical regions and minimized artefacts and noise sources for midbrain and brainstem regions (see the section “Acquisition, reconstruction, and preprocessing of MRI data” in Materials and Methods).

**Fig. 5.**
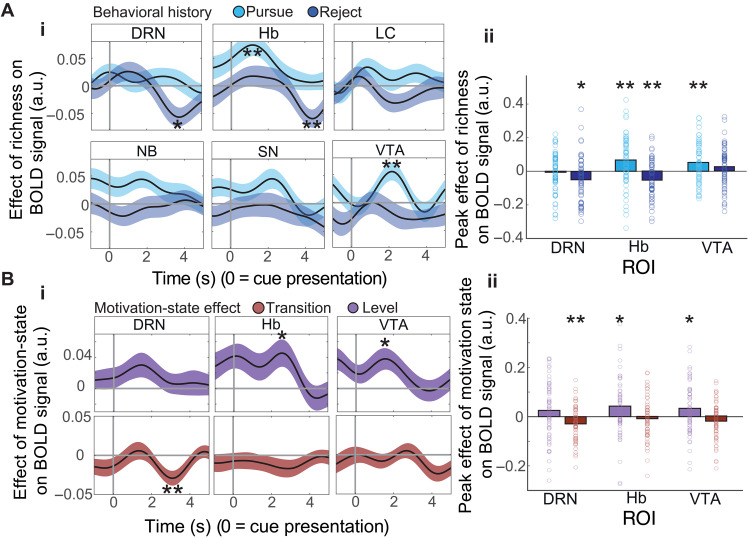
Brain activity in DRN represents the richness of an animal’s environment and transitions between motivation-states. (**A-i**) Time course of the effect that the richness of the environment (β-richness) has on BOLD signal in subcortical ROIs. Time (*x* axis) depicted relative to the onset of reward opportunities when pursue-versus-reject decisions are made (i.e., time = 0 corresponds to decision time). BOLD signal in macaque monkeys peaks approximately 2 to 4 s after neural activity. The timing of peak β weights in DRN, VTA, and Hb is therefore consistent with brain activity that occurs at the time of decision-making. We did not find an effect of environment richness as a function of behavioral history in any other ROI (all *P*s > 0.05). The panel shows the analysis separately as a function of behavioral history (GLM3.2; see Results for explanation). (**A-ii**) Distribution of peak effect sizes of richness of the environment (β-richness) on BOLD signal. Dots indicate peak effect sizes for individual sessions. Separating the data in this way revealed notable patterns in the representation of the environment across regions. DRN represented the richness of the environment with negative sign after the previous opportunity was rejected, VTA represented the richness of the environment with positive sign when animals pursued the previous opportunity, and Hb exhibited distinct positive and negative signals on trials after pursuits and rejections, respectively. (**B-i**) Time course of the effect of motivation-state level (high- versus low-motivation state, purple) and motivation-state transitions (red) on BOLD activity in ROIs that represented the reward environment. (**B-ii**) Distribution of peak effect sizes for motivation-state level (purple) and motivation-state transitions (red) on brain activity. Only DRN represents transitions between motivation-states. Dots indicate peak effect sizes for individual sessions. *P < 0.05, **P < 0.01, ***P < 0.001.

We first examined brain activity during the pursue-versus-reject decision made on each trial (GLM3.2; Materials and Methods). We focused on activity representing the richness and stochasticity of the reward environment at this time. Note that the richness of the environment was correlated with an animal’s behavioral history (see the section “Animal behavior is modulated by the environment”). We therefore performed our analysis separately for trials in which the previous encounter was (i) pursued and (ii) rejected to ensure that any patterns of brain activity that we identified were specific to the richness of the environment, and not related to behavioral history. We note, however, that the key aspects of DRN’s relationship with the richness of the environment obtain regardless of whether the data are analyzed separately in this way (Fig. 5A).

DRN activity was negatively correlated with the richness of the environment after rejection of opportunities [one-sample two-tailed *t* test; GLM3.2; *t*_DRN;rejected_(58) = −2.80, *P* = 0.034; *t*_DRN;pursued_(58) = −0.37, *P* = 0.713 after Bonferroni correction for multiple comparisons, as are all subsequent *t* tests; Fig. 5A, see legend for further discussion of timing of BOLD signal]. In contrast, VTA activity was prominent following opportunity pursuits [GLM3.2; *t*_VTA;pursued_(58) = 3.30, *P* = 0.009; *t*_VTA;rejected_(58) = 1.60, *P* = 0.350; Fig. 5A], and Hb exhibited aspects of both DRN and VTA patterns [GLM3.2; *t*_Hb;pursued_(58) = 3.32, *P* = 0.009; *t*_Hb;rejected_(58) = −3.37, *P* = 0.008; *t*_Hb;rejected-versus-pursued_(58) = 4.95, *P* < 0.001; Fig. 5A]. A two-way analysis of variance (ANOVA) followed by pairwise comparisons confirmed that the effects in DRN and VTA after rejections and pursuits, respectively, were different from one another but not from the corresponding Hb effect [*F*_ROI_(2, 348) = 8.106, *P* < 0.001, *F*_Behav.-history_(1, 348) = 22.305, *P* < 0.001; *F*_ROI-by-Behav.-history_(2, 348) = 4.521, *P* = 0.012; *t*_VTA versus DRN;pursued_(58) = 2.501, *P* = 0.015; *t*_DRN versus VTA;rejected_(58) = −3.403, *P* < 0.001; *t*_VTA versus Hb;pursued_(58) = 0.650, *P* = 0.518; *t*_DRN versus Hb;rejected_ (58) = −0.214, *P* = 0.831]. We did not find an effect of environment richness as a function of behavioral history in any other ROI (all *P*s > 0.05). Despite previous evidence linking DRN with reward uncertainty, we found no relationship between the environment’s stochasticity and brain activity in DRN [GLM3.1; *t*_DRN_(58) = 1.10, *P* = 0.824] and no evidence that stochasticity was represented in other ROIs (GLM3.1; see fig. S11).

After determining that DRN, VTA, and Hb were the key regions tracking the environment, we tested whether they were linked to the motivation states identified with the GLM-HMM. We first quantified the relationship between brain activity and motivation-state level (high versus low). This showed that high motivation states were signaled by increased activity in VTA and Hb but not in DRN [GLM3.4; *t*_Hb;motivation state_(58) = 2.34, *P* = 0.045; *t*_VTA;motivation state_(58) = 2.82, *P* = 0.019; *t*_DRN;motivation state_(58) = 1.75, *P* = 0.084; Fig. 5B]. We next examined activity related to motivati on-state transitions (change versus no change) by identifying transition trials on which the maximum a posteriori motivation state was different from the preceding trial. For each transition trial, we defined a corresponding transition period comprising a symmetric seven-trial window centered on the transition trial (i.e., the transition trial ± three trials). We did this for two reasons: (i) The biological process of transitioning between motivation states is likely to unfold over time instead of occurring instantaneously, meaning that brain activity related to such transitions will occur over a series of trials. (ii) Validation of the GLM-HMM on simulated data showed that state transitions could be accurately decoded within a ± three-trial window (see Fig. 4C). On average, these transition periods comprised 35% of the total trials in a given session. Brain activity in these periods showed a notable dissociation whereby DRN—but neither Hb nor VTA—signaled transitions between motivation states [GLM3.4; *t*_DRN;state transition_(58) = −3.211, *P* = 0.006; *t*_Hb;state transition_(58) = −1.43, *P* = 0.160; *t*_VTA;state transition_(58) = −1.80, *P* = 0.152; Fig. 5B; see fig. S14 (B and C) for data from individual animals].

**Fig. 6.**
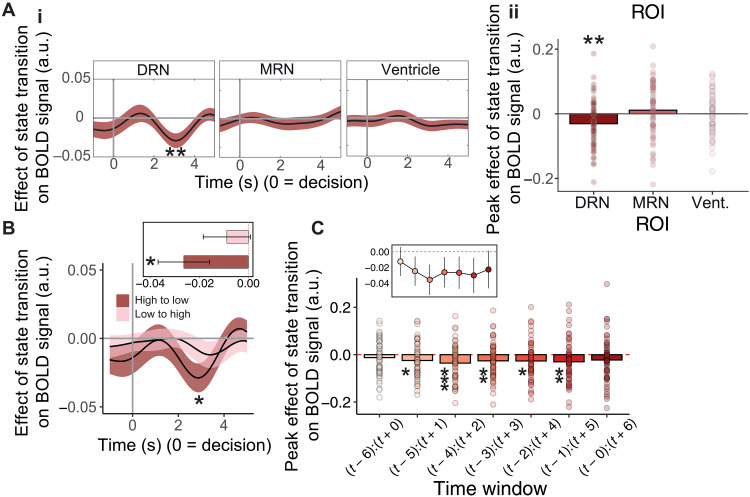
Motivation-state activity in DRN is spatially and temporally specific. (**Ai-ii**) Motivation-state transitions affected BOLD signal in DRN but not neighboring features like MRN and the 4th ventricle, confirming that the pattern was DRN specific. (**B**) The time course of the effect that low-to-high (pink) and high-to-low (red) motivation-state transitions have on DRN BOLD activity. The effect is strongest for high-to-low state transitions. Inset panel shows distribution of peak effect sizes. (**C**) We initially tested the effect of motivation-state transitions on brain activity in a symmetric seven-trial window centered on the transition trial (i.e., the transition trial ±3 trials). This indicated that transitions were represented in the BOLD activity of DRN (Fig. 5B). We confirmed that this effect was not an artefact of our initial window selection by iterating the analysis over different seven-trial windows comprising transitions events. This showed that the effect was robust to window position [*t*_(*t*–5):(*t*+1)_(58) = −2.63, *P* = 0.011; *t*_(*t*–4):(*t*+2)_(58) = −3.69, *P* < 0.001; *t*_(*t*–3):(*t*+3)_(58) = −2.70, *P* = 0.009; *t*_(*t*–2):(*t*+4)_(58) = −2.60, *P* = 0.012; *t*_(*t*–1):(*t*+5)_(58) = −2.69, *P* = 0.009; *t*_(*t*–0):(*t*+6)_(58) = −1.88, *P* = 0.065; GLM3.6]. Note that the windows featuring the least overlap with decoded motivation transitions show nonsignificant effects [*t*_(*t*–6):(*t*+0)_(58) = −1.34, *P* = 0.180; *t*_(*t*–0):(*t*+6)_(58) = −1.88, *P* = 0.065]. Inset (C) shows mean ± 95% CI of transition effect across time windows. *x* axis indicates position of transition period relative to decoded transition time (where *t* = 0 represents decoded transition trial). In time course graphs (A-i and B), lines and shadings show the mean and SE of the β weights across the sessions, respectively. Effect-size graphs (A-ii and C) show size of the peak regression weight in the time course selected using an unbiased leave-one-out procedure (see Materials and Methods). Dots indicate peak regression weight from individual sessions. Error bars in (B) inset show SD of session-wise regression weights. *P < 0.05, **P < 0.01, ***P < 0.001.

The motivation-state transition effect was localized to DRN: Analyzing BOLD signal from adjacent anatomical features, such as the medial raphe nucleus (MRN) and fourth ventricle, showed that there were no corresponding patterns of activity in these areas, and the motivation-state effect in DRN was, indeed, greater than the null effects in MRN and the fourth ventricle [Fig. 6A; *t*_DRN_(58) = −3.21, *P* = 0.006, *t*_MRN_(58) = 1.01, *P* = 0.638, *t*_Vent_(58) = −0.007, *P* = 0.994, *t*_DRN-versus-MRN_(58) = −3.13, *P* = 0.003; *t*_DRN-versus-Vent_(58) = −2.21; *P* = 0.031]. We then probed the temporal specificity of the DRN activity pattern by varying the position of the transition periods examined in the fMRI analysis relative to the transition trials identified by the GLM-HMM. Activity changes in DRN were most prominent when the analyzed period was closely aligned with the transition trial decoded by the GLM-HMM, suggesting that they were indeed specific to motivation-state transition events (Fig. 6C; see fig. S14A for comparison between analysis periods that include and exclude the transition trial). Last, we parsed motivation-state transitions according to direction (i.e., high to low and low to high). This indicated that DRN activity covaried with high-to-low transitions but not low-to-high transitions [GLM3.4 *t*_DRN;high-to-low transitions_(58) = −2.51, *P* = 0.015; *t*_DRN;low-to-high transitions_(58) = −0.91, *P* = 0.365; Fig. 6B]. Together, these analyses suggested that DRN—and DRN specifically—implemented negative changes in intrinsic motivation for rewards.

### Noninvasive disruption of DRN perturbs motivation-state transitions

**Fig. 7.**
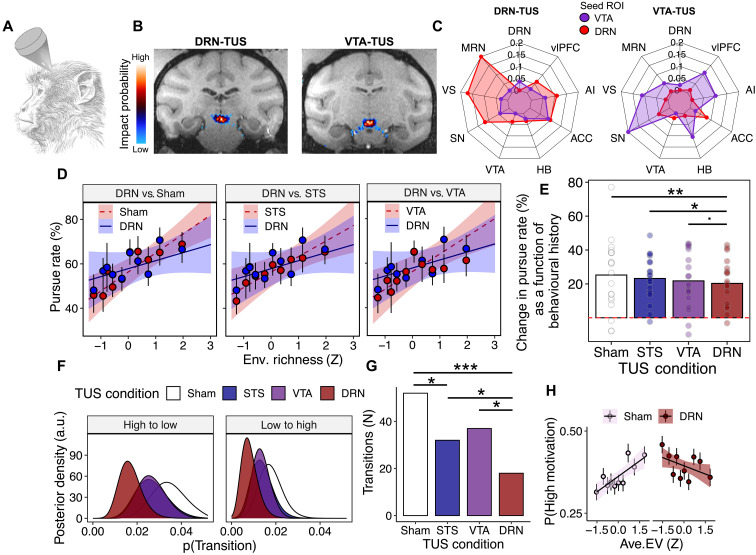
DRN is causally involved in the relationship between the environment and behavior. (**A**) We investigated the causal contribution of DRN to behavior with TUS. (**B**) Simulation of ultrasound propagation under the TUS protocol suggested target-specific sonication after DRN-TUS and VTA-TUS. Color scale indicates the probability of neuromodulation after bilateral DRN/VTA TUS (see fig. S15). The low impact probability corresponds to 0.5 MPa. High impact probabilities at each acoustic focus are shown in fig. S15. (**C**) DRN-TUS disrupted DRN (red) connectivity with key brain regions while leaving VTA (blue) connectivity intact. The opposite pattern occurred after VTA-TUS. Radial axis shows absolute difference in connectivity between seed and target ROIs pre-versus-post TUS (see Materials and Methods). Targets included vlPFC, AI, ACC, Hb, SN, VERSUS, MRN, and DRN. (**D**) DRN-TUS attenuated the richness of the environment’s effect on behavior relative to sham-TUS, STS-TUS, and VTA-TUS. Points and whiskers indicate means (±SEM) pursue rate across deciles of environmental richness. (**E**) DRN-TUS attenuated behavioral history’s effect on current behavior relative to sham-TUS and STS-TUS, and marginally compared to VTA-TUS. *y* axis indicates difference in pursue rate based on behavioral history (pursue-rate_behav.history==pursue_ – pursue-rate_behav.history==reject_). Positive values indicate that animals were more likely to pursue a reward if they also pursued the previous one. Points indicate differences in mean pursue rates per session. (**F**) Posterior probability of state transitions in GLM-HMMs fitted to each TUS condition. The central tendency of DRN-TUS posteriors is lower than STS-TUS, VTA-TUS, and sham-TUS posteriors. (**G**) Fewer motivation-state transitions occurred in DRN-TUS sessions versus all other TUS conditions. Data shown as sum of all motivation-state transitions in each TUS condition. (**H**) DRN-TUS diminished the relationship between the average value of recent opportunities and motivation-state level. Dots and whiskers indicate means (±SEM) level of high-motivation state occupancy in deciles of average expected value (Ave. EV). *P < 0.05, **P < 0.01, ***P < 0.001.

fMRI recordings linked DRN to behavior in two complementary ways; (i) DRN coded the richness of the environment, which modulated an animal’s pursue-versus-reject decisions toward specific reward opportunities, and (ii) DRN coded transitions between motivation states, and therefore changes in reward pursuit over multi-trial timescales. We tested DRN’s causal contribution in these respects with a second experiment using TUS (Fig. 7A)—a minimally invasive and reversible technique that disrupts brain activity via kinetic interactions between focused ultrasound waves and the neuron and astrocyte membrane that, in turn, induce changes in adjacent synapses (*29*, *30*). As a result, short TUS trains of the type we used are known to produce short-term changes in neural activity by inducing *N*-methyl-d-aspartate–dependent plasticity (*31*–*35*) leading to changes in activity in spatially circumscribed gray brain regions that are targeted. As a result, a region targeted with TUS alters its responsiveness to activity in interconnected areas, while nonstimulated areas show no such change (*17*).

We used an offline TUS protocol that modulates neural activity for over an hour after sonication (*17*, *36*, *37*). We compared DRN-TUS to three control conditions: a sham condition (sham-TUS), an active cortical control (superior temporal sulcus; STS-TUS), and an additional active subcortical control in VTA. We chose VTA as a subcortical control region because it was implicated in motivation states during fMRI recordings in distinct ways to DRN, thereby allowing us to test the specificity of DRN’s function. We chose STS as our active cortical control condition because it is a region closest to the transducer position when TUS is targeted to DRN and through which the ultrasound beam might pass when targeting the DRN. It is therefore ideally located to rule out any potential side effect of sound beams on brain structures due to some process of reflection or refraction from skull.

We first performed simulations of acoustic wave propagation under the TUS protocol, which established that selective perturbation of DRN and VTA was feasible despite their diminutive size and anatomical location (Fig. 7B and fig. S15). We then empirically tested the TUS protocol’s anatomical specificity by measuring changes in functional connectivity between DRN, VTA and a series of key brain areas before-versus-after DRN-TUS and—as a control—before-versus-after VTA TUS. This showed notable and selective changes in region-specific functional connectivity: DRN-TUS disrupted DRN’s ordinary patterns of coactivation with key interconnected regions but left VTA connectivity unchanged (Fig. 7C; see also fig. S18). Conversely, after VTA-TUS, VTA connectivity changed but DRN connectivity did not (Fig. 7C). In combination with the sonication simulations, this indicated that TUS induced safe, efficacious, and highly localized disruption of DRN and VTA activity, analogous to previous investigations of TUS in cortical brain regions (*17*, *36*, *37*).

We tested the same monkeys from the fMRI experiment on five sessions of the behavioral task per TUS condition in counterbalanced and pseudorandomized order (see Materials and Methods). We first investigated how TUS modulated the influence of (i) richness of the environment and (ii) behavioral history, which were the key factors driving the pursue-versus-reject decisions made on each trial. DRN-TUS diminished the effect of richness of the environment relative to all other conditions (GLM4.1; β_DRN-TUS versus sham-TUS*richness_ = −0.18, SE = 0.05, *P* < 0.001; β_DRN-TUS versus STS-TUS*richness_ = −0.12, SE = 0.06, *P* = 0.025; β_DRN-TUS versus VTA-TUS*richness_ = −0.12, SE = 0.06, *P* = 0.035; Fig. 7D). Similarly, DRN-TUS clearly reduced the effect of behavioral history relative to sham-TUS and STS-TUS and produced a marginal difference relative to VTA-TUS (GLM4.2; β_DRN-TUS versus sham-TUS*behave-history_ = −0.42, SE = 0.11, *P* < 0.001; β_DRN-TUS versus STS-TUS*behave-history_ = −0.24, SE = 0.11, *P* = 0.027; β_DRN-TUS versus VTA-TUS*behave-history_ = −0.20, SE = 0.11, *P* = 0.057; Fig. 7E). There were no differences between active control conditions (STS-TUS and VTA-TUS) and sham-TUS (GLM4.1; Fig. 7, D and E; β_VTA-TUS versus sham-TUS*richness_ = −0.06, SE = 0.06, *P* = 0.282; β_STS-TUS versus sham-TUS*richness_ = −0.06, SE = 0.05, *P* = 0.306; GLM4.2; Fig. 7E; β_VTA-TUS versus sham-TUS*behavioral-history_ = −0.20, SE = 0.10, *P* = 0.060; β_STS-TUS versus sham-TUS*behavioral-history_ = −0.18, SE = 0.11, *P* = 0.090). DRN, therefore, was specifically and causally involved in the impact of an animal’s recent reward environment and behavior on its present decisions to pursue reward.

Next, we applied the GLM-HMM to behavioral data from the TUS experiment (see Materials and Methods). Our analysis focused on motivational-state transitions, which were a distinguishing feature of DRN activity in fMRI recordings. These recordings suggested that DRN was specifically involved in high-to-low motivational-state transitions, but we first performed the analysis in a direction-neutral way given the interdependence of time-series observations: Because the animals typically began the task in a high-motivation state [*P*(init-state = high) = 0.70], reducing the likelihood of high-to-low transitions would necessarily reduce the frequency of low-to-high transitions in a fixed-length time series, even if the underlying low-to-high transition probability was unchanged. The analysis showed a notable pattern whereby DRN-TUS reduced the likelihood of transitions relative to all other TUS conditions (GLM4.3; β_DRN-TUS versus sham-TUS_ = −1.08, SE = 0.27, *P* < 0.001; β_DRN-TUS versus VTA-TUS_ = −0.71, SE = 0.29, *P* = 0.013; β_DRN-TUS versus STS-TUS_ = −0.57, SE = 0.29, *P* = 0.051; Fig. 7, F and G). There was no effect of VTA-TUS relative to sham-TUS (GLM4.3; β_VTA-TUS versus sham-TUS_ = −0.37, SE = 0.22, *P* = 0.090). There was a moderate difference between STS-TUS and sham-TUS, but this was outweighed by the fact that DRN-TUS reduced the likelihood of transitions even relative to STS-TUS (GLM4.3; β_STS-TUS versus sham-TUS_ = −0.51, SE = 0.23, *P* = 0.022; β_DRN-TUS versus STS-TUS_ = −0.57, SE = 0.29, *P* = 0.051). DRN-TUS was, moreover, the only condition that reduced high-to-low transitions specifically (GLM4.4; β_DRN-TUS versus sham-TUS_ = −0.77, SE = 0.32, *P* = 0.016; β_VTA-TUS versus sham-TUS_ = −0.33, SE = 0.28, *P* = 0.238; β_STS-TUS versus sham-TUS_ = −0.43, SE = 0.29, *P* = 0.139). Last, DRN-TUS caused animals to spend more time in high-motivation states relative to sham-TUS (GLM4.5; β_DRN-TUS versus sham-TUS_ = 0.28, SE = 0.07, *P* < 0.001), consistent with the idea that DRN-TUS prevented animals from transitioning to low-motivation states. Note that motivation-state transitions are rare events, regardless of the TUS condition. However, our analysis clearly shows that DRN disruption significantly reduces or even eliminates these events, compared to other conditions (see fig. S16 for data from individual animals).

Last, we sought to specify how DRN controlled transitions between motivation states. To do so, we returned to an earlier analysis demonstrating that motivation states were matched to the availability of rewards: In brief, animals were more likely to occupy high-motivation states when there were many high-value rewards available, analogous to the way that animals pursued more opportunities in rich environments (Fig. 4H). Given DRN’s critical involvement in the latter phenomenon, we asked whether its control of motivational-state transitions was mediated by the availability of rewards. Consistent with this view, DRN-TUS diminished the relationship between the availability of rewards and motivation states relative to all other TUS conditions (GLM4.5; Fig. 7H; β_DRN-TUS versus sham-TUS*Ave.EV_ =−0.28, SE = 0.07, *P* < 0.001; β_DRN-TUS versus STS-TUS*Ave.EV_ = −0.29, SE = 0.07, *P* < 0.001; β_DRN-TUS versus VTA-TUS*Ave.EV_ = −0.30, SE = 0.07, *P* < 0.001). There were no effects of VTA-TUS or STS-TUS relative to sham-TUS (β_VTA-TUS versus sham-TUS*Ave.EV_ = −0.03, SE = 0.06, *P* = 0.610; β_STS-TUS versus, sham-TUS*Ave.EV_ = −0.05, SE = 0.07, *P* = 0.499). Intriguingly, DRN-TUS’s influence was more prominent for the link between low-value reward environments and low-motivation states, which dovetailed with fMRI signals implicating DRN specifically in high-to-low transitions (GLM4.5; for Ave. EV < μ_Ave.EV_ β_DRN-TUS*Ave.EV_ = −0.52, SE = 0.19, *P* = 0.006; for Ave. EV ≥ μ_Ave.EV_ β_DRN-TUS*Ave.EV_ = −0.13, SE = 0.15, *P* = 0.403). Together, these results suggest that DRN has a fundamental role in ensuring that an animal’s motivation state is appropriate to the distribution of rewards in the environment.

### A cortico-subcortical circuit reconciles behavior with the environment

In a final analysis, we examined interactions between DRN and other brain regions that might relate to its behavioral function. To do so, we first reverted to the analysis of brain activity and behavior that did not rely on the GLM-HMM framework (Fig. 5A). Here, DRN, VTA, and Hb represented an animal’s environment with complementary patterns of activity, which suggested that they might form a circuit for reconciling decisions with the surrounding environment. To test the circuit hypothesis, we expanded our purview to functionally related cortical ROIs in supplementary motor area (SMA), anterior cingulate cortex (ACC), and anterior insula (AI) based on previous work implicating these regions in behavioral change (*9*, *36*, *38*–*40*). Only AI activity signaled the richness of the environment with the distinctive contingency on behavioral history seen in subcortical ROIs, and we therefore retained AI as a cortical ROI for connectivity analysis (see fig. S13).

**Fig. 8.**
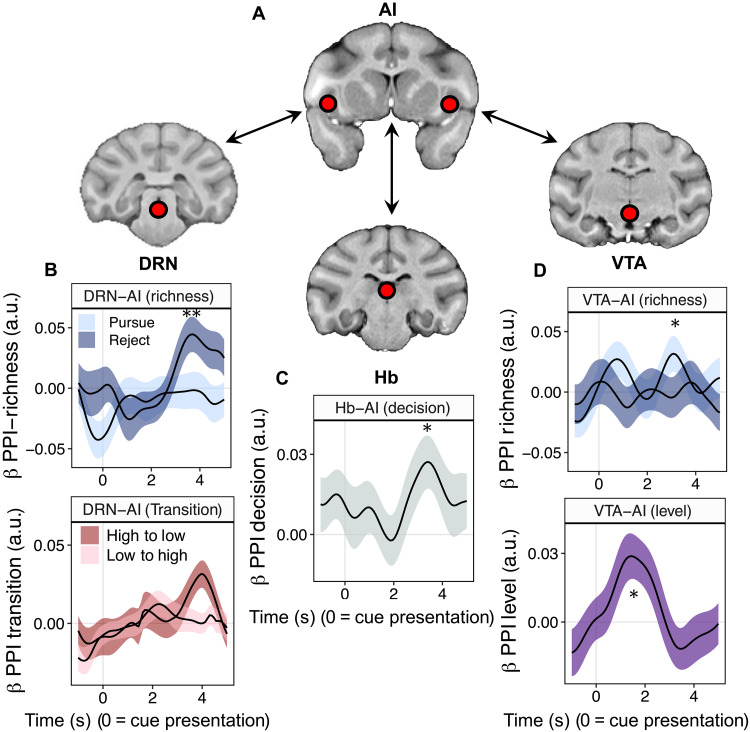
A brain circuit for reconciling an animal’s motivation with its environment. (**A**) PPIs indicated the following patterns of cortico-subcortical connectivity. (**B**) Connectivity between DRN and AI increased as a function of richness of the environment in the aftermath of rejections (GLM3.6; top), and as a function of high-to-low motivation-state transitions [bottom; GLM3.7; *t*_PPI(high-to-low)_ = 3.64, *P* < 0.001], consistent with DRN’s fMRI signals (**C**) connectivity between AI and Hb increased as a function of an animal’s ultimate decision about whether to pursue a reward opportunity (GLM3.8). (**D**) In contrast to DRN, connectivity between VTA and AI increased as a function of richness of the environment in the aftermath of pursuits (GLM3.6; top) and during high-motivation states (GLM3.7; *t*_motivation-level_ = 2.47, *P* = 0.016). *P < 0.05, **P < 0.01, ***P < 0.001.

Next, we asked which ROIs coded the pursue-versus-reject decision taken on each trial—in other words, which ROIs might be the output of the putative decision-making circuit. This demonstrated that BOLD activity time-locked to decision-making in both Hb and AI represented pursue-versus-reject decisions [GLM3.3; *t*_pursue;Hb_(58) = 3.24, *P* = 0.011; *t*_pursue;AI_(58) = 2.78, *P* = 0.028; fig. S12]. We then conducted psychophysiological interaction (PPI) analyses to probe changes in pairwise connectivity between regions as a function of the environment (*41*). PPIs mirrored the patterns observed earlier: Connectivity between DRN and AI increased as a function of richness of the environment after rejections [GLM3.6; *t*_PPI(DRN-AI by richness);rejected_(58) = 2.84, *P* = 0.006; Fig. 8B] and increased between VTA and AI after pursuits [GLM 3.6; *t*_PPI(AI-VTA by richness);pursued_(58) = 2.14, *P* = 0.036; Fig. 8D]. Adopting the GLM-HMM analysis approach showed a corresponding pattern of results (GLM3.7; Figs. 8B and 6A-iii). Connectivity between Hb and AI was not modulated by the richness of the environment but instead by the pursue/reject decision taken on each trial, consistent with a role in translating motivation to action (*42*) [GLM3.8 *t*_PPI(AI-Hb by action)_(58) = 2.25, *P* = 0.028; Fig. 8C].

## DISCUSSION

We provide converging evidence that DRN controls changes in an animal’s motivation for rewards. We observed distinctive patterns of DRN activity corresponding to the richness of an animal’s environment and transitions between statistically delineated motivation states. We followed this with minimally invasive DRN disruption, which diminished the environment’s effect on decision-making and reduced the frequency of motivation-state transitions. This suggests that DRN is causally involved in transitions between a high-motivation state occupied when rewards are abundant and a low-motivation state engendered by reward scarcity.

We used a GLM-HMM approach to describe features of animal behavior that are difficult to capture with a standard GLM. In particular, we tested the hypothesis that animals adopted different motivation states using a GLM-HMM in which only the bias parameter changed between HMM states. This model allowed us to identify periods where animals were more likely—all else equal—to pursue rewards, and periods where animals were less likely—all else equal—to pursue rewards, and to identify points of transition between these periods. The number of trials in our dataset did not allow us to implement more complicated versions of the GLM-HMM where, for example, the transition matrix is modeled as a function of covariates instead of as fixed parameters (*43*). A GLM-HMM with a covariate-dependent transition matrix would be an interesting subject for future research. Similarly, we acknowledge that other descriptive and/or mechanistic models would likely reveal further interesting features of behavior (*44*–*46*). Here, however, we focused on the specific hypothesis that animals would experience shifts in the baseline likelihood of pursuing rewards.

**Fig. 9.**
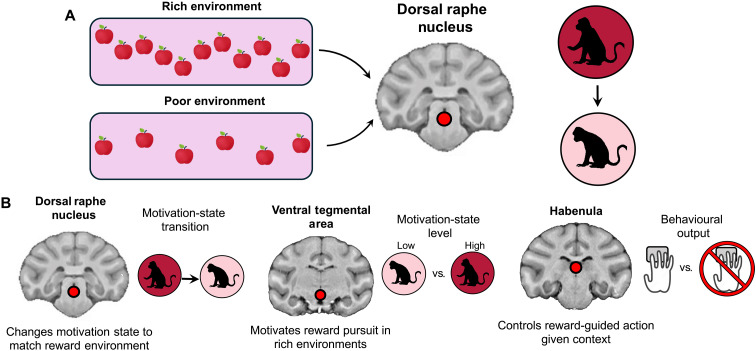
DRN controls motivation-state transitions as a function of the environment. (**A**) We provide converging evidence from recoding and disruption techniques that DRN controls transitions between high-motivation states occupied when rewards are abundant, to low-motivation states engendered by reward scarcity. We started by showing that the purse-versus-reject decision made on each trial was influenced by an animal’s recent history of reward (richness of the environment) and previous pursue-versus-reject behavior (behavioral history) on (Fig. 1, D to G). These effects indicated that behavior was autocorrelated, consistent with the idea that animals occupied an underlying hidden motivation state that could not be explained solely by the external environment (Fig. 2A). To test this hypothesis, we used a GLM-HMM framework. We identified state-like fluctuations in motivation to pursue rewards (Fig. 2, C to E) that were discrete, persistent, and biologically meaningful (Fig. 4). Using an optimized fMRI approach, we then identified three nuclei (DRN, HB, and VTA) that tracked animals’ external environment and GLM-HMM–derived motivation states. HB and VTA encoded the level of motivation state (whether animal was in a high or low motivation-state; Fig. 5B). DRN specifically encoded transitions between high-to-low motivation states (Fig. 5B). Last, we complemented the fMRI results with a causal intervention. We showed that disrupting DRN perturbed the previously observed effects of the external environment on behavior (Fig. 7, D and E) and, importantly, reduced the likelihood of transitions in motivation-states (Fig. 7, F to H). (**B**) We thereby distinguish DRN’s function from interconnected subcortical nuclei, which are also implicated in motivation. In combination with previous studies, we suggest a complementary scheme in which DRN (left) controls transitions between high-to-low motivation states, VTA (middle), implements and maintains the changes in reward sensitivity that characterize motivation states, and habenula (right) integrates an animal’s internal and external context to control its behavioral output.

Our findings dovetail with two prevailing views on DRN function: The first has emphasized DRN’s role in behavioral changes, including between exploration and exploitation or patience and impulsivity (*5*, *47*, *48*). For example, the firing rate of DRN serotonin neurons increase during waiting for delayed reward (*49*). The second has linked DRN activity to statistical aspects of rewards, like the general value and/or uncertainty of the options characterizing an environment (*6*–*10*, *50*). We observed similar brain-behavior relationships here, and the co-occurrence of both phenomena suggests that they are fundamentally related. In support of this view, we found that DRN disruption impaired animals from matching their motivation state with the availability of rewards, and specifically from entering low-motivation states during low-value environments, which was an important motif in their normal behavior. In tandem with previous studies, this indicates that DRN is critical for reconciling an animal’s behavior with the reward statistics of the surrounding world (Fig. 9). An alternative interpretation of the DRN disruption effect is that fewer transitions are decoded because the two motivation states were more similar to one another. Although the motivation states identified during DRN TUS sessions were moderately more similar to one another than the motivation states identified in other conditions (see fig. S17), it is important to emphasize that the animals clearly exhibited two discrete motivation states after DRN TUS and that fewer transitions between states occurred in the DRN TUS condition (Fig. 7, F and G). Furthermore, the motivation-state transition interpretation is consistent with the correlation between transition events and DRN activity observed in fMRI recordings (Figs. 5B and 6). We found no evidence that DRN activity was related to motivation-state level (high versus low; Fig. 5B), which one would expect if DRN’s role was to maintain or distinguish the motivation-states themselves.

Several previous studies have identified DRN activity in tasks where there is fundamental uncertainty about the reward available on each trial (*3*, *51*, *52*). In contrast, the properties of specific reward opportunities were explicitly signaled in our paradigm, and the key manipulation was the distribution of reward opportunities over time. This might explain the absence of reward-uncertainty signals in DRN fMRI recordings. It might similarly explain VTA’s involvement in the task. Although it is perhaps unexpected that VTA did not feature more prominently given its well-documented links to reward and motivation, the task did not require reinforcement learning, which is a key function of dopaminergic nuclei (*53*). Instead, we observed patterns of VTA activity corresponding to (i) the richness of the environment after pursuits and (ii) trial-by-trial motivation-state level, which suggest a cognate role in driving behavior in proximity to rewards (Fig. 9) (*23*).

How might DRN coordinate with other regions of the primate brain to control decisions? Unlike other subcortical ROIs, we found that activity in Hb covaried with both an animal’s pursue/reject decisions and the individual factors that shaped them. This is consistent with an emerging theory that Hb integrates motivationally salient information from its diverse range of afferent connections to control motor output via the basal ganglia (Fig. 9) (*42*, *54*–*56*). Similar patterns of activity occurred in AI, which is perhaps the computational source of representations concerning rewards obtained over multiple time points (*9*, *42*, *57*). Although it is difficult to specify directions of influence from fMRI recordings, we observed patterns of functional connectivity consistent with communication between AI-DRN, AI-VTA, and AI-Hb—a scheme that matches well-documented patterns of anatomical connection (*58*, *59*). Cortico-subcortical interactions of this kind echo a common neural motif whereby information synthesized in the cortex is transmitted to subcortical nuclei, which have wide-ranging efferent connections capable of orchestrating brain-wide activity (*42*, *55*).

The fact that animals were ceteris paribus more likely to pursue rewards in high-value environments controverts prominent formulations of optimal reward-seeking (*1*), which argue that animals should be less selective during periods of reward scarcity on account of their homeostatic requirements. Nevertheless, the pattern observed ensures that animals maximize their reward intake in the manner that these theories envisage: Given that animals usually lack perfect knowledge about the richness or sparseness of future opportunities, a reasonable strategy is to concentrate their efforts on rich environments where they have the most to gain (*9*, *10*, *23*). In support of this interpretation, the GLM-HMM analysis identified persistent motivation states in monkey behavior that were positively correlated with the availability of rewards.

Last, many neurons in DRN are serotonergic and that DRN is the principal source of serotonin to the mammalian forebrain (*60*, *61*). Although the techniques we implemented are not serotonin specific, our results are consistent with several proposals about its function. We found (i) a positive correlation between DRN activity and reward scarcity, evoking classic theories that link serotonin to punishments and reward omissions (*24*, *62*–*64*), and (ii) a causal relationship between DRN and changes in behavior, akin to serotonin’s role in choice perseveration (*4*, *5*). The key finding in our experiment relative to previous work is that DRN controlled patterns of behavior that unfolded over multiple time points and not just specific reinforcement events or decisions. In a similar vein, it is possible that serotonin is important not for moment-to-moment decision-making per se but for changing the overarching strategy of an animal’s behavior and perhaps especially for changes that involve adverse feedback from the environment. Testing this hypothesis is a promising avenue for future experiments.

## MATERIALS AND METHODS

### Experimental model and subject details

The experiments were performed with four male rhesus macaques (*M. mulatta*). The sample size corresponded to those used in previous studies in which it had been possible to identify significant and reliable whole-brain fMRI recording and TUS across multiple testing sessions but with the minimum number of animals. The animals were 10 to 12 years of age and weighed 14.30 to 17.09 kg. They lived in group housing with a 12-hour light-dark cycle and were afforded access to water for 12 to 16 hours on testing days and free access on nontesting days. All procedures were conducted under a license issued by the UK Home Office in accordance with the Animal (Scientific Procedures) Act 1986 and the European Union guidelines (EU Directive 2010/63/EU).

### Behavioral training

All monkeys were fitted with MRI compatible cranial implants that facilitated head fixation during testing and training. Training was conducted in MRI compatible chairs designed to accommodate monkeys in a sphinx position and took place in custom-built environments that replicated an MRI scanner. The animals were trained on simplified versions of the task in which they did not need to wait go-cue and action-outcome delays and were not exposed to controlled changes in their reward environment. Go-cue and action-outcome delays were then introduced and carefully increased over the course of training until they were sufficient for an fMRI experiment (see below). Changes in the reward environment were introduced after the animals were accustomed to the final delay timings. Testing for both the fMRI and TUS experiments began when the proportion of pursued trials per session was stationary over consecutive days.

### Behavioral task

Animals performed a simple decision-making task involving sequential encounters with reward opportunities that appeared on a computer screen. Reward opportunities were presented in visual form via colored boxes (size of the box: 8 cm by 26 cm) that were filled with dots. The color of the stimulus indicated the number of juice drops that the opportunity was worth (red = 1; green = 2; blue = 3). The number of dots comprising the stimulus indicated the probability that reward would be delivered if the opportunity was pursued. Reward probabilities ranged between (0.05, 1), and dots indicated 0.05 linear increments of reward probability. The stimuli were designed so that dots gradually filled the box from the top downward.

Opportunities first appeared in the center of the screen before displacing either right or left (Fig. 1A). The displacement of the opportunity stimulus functioned as a go cue, which indicated that the opportunity was available for pursuit. Monkeys could pursue opportunities by manually responding to an infrared sensor corresponding to the opportunity’s on-screen location—For example, if the opportunity displaced right, they needed to touch a sensor with their right hand, and vice versa if it displaced left. The go cue was designed to temporally dissociate decisions about pursuing opportunities from the motoric processes, which realized decisions in behavior. Similarly, the randomized right/left displacement of the opportunity prevented monkeys from motor planning during the decision phase. The durations separating opportunity onsets and go cues were drawn from *go-cue* ~ uniform(3, 4) and were optimized for a canonical macaque hemodynamic response function (HRF) of approximately 4 s. Monkeys had 1 s to pursue opportunities after the go cue. After pursuing opportunities, the monkeys needed to wait action-outcome delays spanning *A-O-delay* ~ uniform(3, 4) before receiving potential juice rewards accompanied by visual reward feedback. The next trial then began after an inter-trial interval (ITI) spanning *ITI* ~ N(1, 4). If the monkeys did not pursue an opportunity, they bypassed the action-outcome and reward-delivery durations and proceeded to the next trial. If the monkeys pursued opportunities before the go cue—i.e., prematurely—they needed to wait for the remaining duration of the opportunity presentation, in addition to go-cue, action-outcome, and reward-feedback durations. The opportunity that elicited the premature response was then repeated on the next trial.

Each session of the task comprised four blocks of 40 to 50 trials. Blocks were used to engender different reward environments by systematically controlling the reward-probability aspect of opportunities. Environments varied on two dimensions: (i) Richness was defined by the mean of the reward-probability distribution, where μ_rich_ = 0.80 and μ_poor_ = 0.55, and (ii) stochasticity was defined by the width of reward-probability distributions, where predictable environments were generated from Gaussian distributions with σ_predictable_ = 0.05 and unpredictable environments from random-uniform distributions with ranges of 0.40 such that σ_unpredictable_ = 0.13. Blocks were characterized along both richness and stochasticity dimensions, which yielded four distinct environment types—rich-predictable, rich-stochastic, poor-predictable, and poor-stochastic. Each session featured one of each environment-type, and the order of environments was counterbalanced with respect to the richness dimension to avoid long periods of low-value offers (e.g., during consecutive poor environments), which were difficult for monkeys to perform. Environment types were indicated by visual cues that bordered the screen and indicated what kind of block the animals were currently in. The visual stimuli were abstract black and white patterns (see the diagonal dashed lines bordering the screen in the diagram of the task in Fig. 1A).

The task was implemented in MATLAB v2019 by MathWorks using Psychophysics Toolbox v3 (*65*) and presented on MRI-compatible screens (23in BOLD screen; Cambridge Research Systems) approximately 30 cm away from the subjects. Juice rewards consisted in a solution of water, blackcurrant cordial, and banana, and each juice drop was 1 ml. Pupillometric data were obtained during fMRI sessions via an MR-compatible infrared EYElink 1000 eye-tracker device by SR Research Ltd. recording pupil diameter and gaze direction along *x* and *y* planes at a 250-Hz sampling rate.

Preprocessing and analysis of pupillometric data were performed in R and entailed the following steps [adapted from (*66*)]. Samples reflecting eye blinks were identified with a detection algorithm native to the EYElink device. Artefacts were defined as consecutive samples with discrepancies of >50 a.u. All samples within symmetric 25 sample (i.e., 0.1 s) windows of artefacts or eye blinks were scrubbed and linearly interpolated, and interpolated time series were then low-pass filtered with a 4-Hz cutoff. These processes were performed for pupil size, *x*-gaze direction, and *y*-gaze direction time series, respectively, which were *z*-scored. We then regressed variance due to *x*-gaze direction and *y*-gaze direction from pupil size via linear regression and performed subsequent analysis using the residuals.

To incorporate pupillometry in our behavioral analysis, we extracted pupil size in specific epochs of the task, with a focus on periods just before and after the presentation of reward opportunities in which animals were making decisions. In particular, we quantified—for each trial—mean pupil size 0.5 to 0 s before (ITI phase) and 0.5 to 1.0 s after (decision phase) stimulus presentation. We did not select the first 0.5 s to avoid pupillometric responses reflecting sudden luminance changes, as when a visual stimulus first appears. When comparing the pupil size between motivation states (GLM2.4;Fig. 4F), we focused on pupil size during the decision phase. On all other occasions, when using it as a confound regressor to control for arousal, we focused on pupil size during the ITI phase. We chose this phase to make sure that it is not confounded by the brightness of the stimulus in front of the animal. Pupil size data were also used to reject periods of behavior, in which animals had their eyes shut (5.1% of the trials on average).

Animals were given four blocks of 40 to 50 trials. They performed one session per day, at the same time of the day.

### Behavioral analysis

Our initial analysis of behavior characterized the influences on binomial pursuit/reject decisions. We accomplished this with mixed-effect binomial GLMs with subject identity as a random variable—This means that all GLMs account for inter-subject variability for the effect of interest. We began by investigating the time horizon of an animal’s sensitivity to past rewards with the following GLM (GLM1.1)

$$\begin{array}{c} \text{logit}\left({\text{decision}}_{t}\right)=\beta_{0}+\beta_{1}{\text{reward-magnitude}}_{t}\\ +\beta_{2}{\text{reward-probability}}_{t}\\ +\beta_{3}\;\left({{\text{reward-magnitude}}_{t}}^{*}{\text{reward-probability}}_{t}\right)\\ +\beta_{4}{\text{reward-outcome}}_{t-1}+\beta_{5}{\text{reward-outcome}}_{t-2}\\ +\beta_{6}{\text{reward-outcome}}_{t-3}\\ +\beta_{7}{\text{reward-outcome}}_{t-4}\\ +\beta_{8}{\text{reward-outcome}}_{t-5}+\beta_{9}{\text{reward-outcome}}_{t-6}\\ +\beta_{10}{\text{reward-outcome}}_{t-7}+\beta_{11}{\text{reward-outcome}}_{t-8}\\ +\beta_{12}{\text{reward-outcome}}_{t-9}+\beta_{13}{\text{reward-outcome}}_{t-10}+\mu_{0}+\varepsilon \end{array}$$

where β_0–13_ are fixed-effects (β_0_ is the intercept), μ_0_ is the by-subject random intercept, and ε is an error term. Decision is animals’ pursue (coded as 1) versus reject (coded as 0) decisions. Reward-magnitude is the reward magnitude of the current opportunity, which varies between one and three drops of juice. Reward-probability is the reward probability of the current opportunity, which varies between 0.35 and 1. Reward-outcome is the actual received reward and varies between zero (no reward), one, two, or three drops of juice. The small number of subjects precluded fitting GLM1.1 with the full random-effects structure—that is, affording all predictors random effects for each subject. To assess the significance of past reward outcomes while also taking inter-subject variability into account, we therefore fit an iterative series of GLMs in which one of the previous outcome predictors was given a random slope (e.g., in the first GLM, reward-outcome_*t*−1_ was given a random slope). We assessed the statistical significance of past outcome regressors using the random effect in the relevant GLM. Figure 1D shows the outcome of this analysis. Given the results from GLM1.1, we operationalized the richness of an animal’s reward environment as a moving average with a retrospective five-trial window, and the stochasticity of its environment as the SD of that reward rate as follows (richness and stochasticity of environment)

$${\text{richness}}_{t}=\text{mean}\left({\text{reward-received}}_{t–5}:{\text{reward-received}}_{t–1}\right)$$

$${\text{stochasticity}}_{t}=\text{sd}\left({\text{richness}}_{t–5}:{\text{richness}}_{t–1}\right)$$

We implemented the following model to assess the factors influencing pursuit/reject decisions (GLM1.2)

$$\begin{array}{c} \text{logit}\left({\text{decision}}_{t}\right)=\beta_{o}+\beta_{1}{\text{reward-magnitude}}_{t}\\ +\beta_{2}{\text{reward-probability}}_{t}+\beta_{3}{\text{environment-richness}}_{t}\\ +\beta_{4}{\text{environment-stochasticity}}_{t}\\ +\beta_{5}\;\left({{\text{reward-magnitude}}_{t}}^{*}{\text{reward-probability}}_{t}\right)\\ +\beta_{6}{\text{trial-number}}_{t}+\mu_{0}+\varepsilon \end{array}$$

where β_0–6_ are fixed-effects, μ_0_ is the by-subject random intercept, and ε is an error term. The small number of subjects precluded fitting GLM1.2 with the full random-effects structure. We therefore fit an iterative series of models in which the key predictors of interest—environment-richness and environment-stochasticity—were individually afforded random effects by animal. All mixed-effects GLMs were performed in R, and models were fit via maximum likelihood estimation as implemented in the lme4 toolbox. We then fit the following GLMs in using the same procedure (GLM1.3)

$$\begin{array}{c} \text{logit}\left({\text{decision}}_{t}\right)=\beta_{o}+\beta_{1}{\text{reward-magnitude}}_{t}\\ +\beta_{2}{\text{reward-probability}}_{t}+\beta_{3}{\text{behavioral-history}}_{t}\\ +\beta_{4}{\text{environment-richness}}_{t}+\beta_{5}{\text{environment-stochasticity}}_{t}\\ +\beta_{6}\left({{\text{reward-magnitude}}_{t}}^{*}{\text{reward-probability}}_{t}\right)\\ +\beta_{7}{\text{trial-number}}_{t}+\mu_{0}+\varepsilon \end{array}$$

where β_0–6_ are fixed-effects, μ_0_ is the by-subject random intercept and ε is an error term.

### Behavioral modeling

We used a GLM-HMM to probe time-varying patterns in behavior that might reflect internal motivation states. A GLM-HMM is an extension of the GLM that consists of two parts. The HMM part of the model assumes that time series events arise from so-called hidden states, which produce observations according to state-dependent probability distributions (*18*, *19*). These states bear Markov relations to one another, such that the state at time *t* is determined strictly by the state at time *t* – 1, and the respective probabilities of transition between states. A simple HMM of animal behavior in this context, for example, might comprise states in which responses arise from binomial distributions with state-dependent probability parameters that reflect changes in an animal’s motivation (i.e., probability of pursuing) across time.

The GLM part of a GLM-HMM parameterizes state-dependent probability distributions according to predictors (*18*). The weights afforded to each predictor can vary from state to state, which enables the model to capture time-varying differences in an animal’s decision-making process. We implemented a GLM-HMM in which pursuit/reject decisions were parameterized by bias terms, in addition to predictors for the expectation value of the specific opportunity animals were faced with on the current trials (cued to the animals by the color of the stimulus and the number of dots comprising the stimulus) and binary environment cues indicating the experimentally manipulated richness and stochasticity of the reward environment (cued to the animals by abstract black and white patterns bordering the screen). We were interested in internal states of motivation—that is, changes in an animal’s intrinsic propensity to pursue rewards. We therefore let only the bias parameter(s) change between states and constrained the weight on expectation value and environment-cue parameters to be the same across states. Formally

$$p\left(y_{t}=\text{pursue}|z_{t}=k,x_{t},w_{k}\right)=\frac{1}{1+e^{-w_{k}\cdot x_{t}}}$$

where *y* ∈ {pursue, reject}^T^ is the animal’s decisions across *T* trials, *z_t_* is the HMM state at trial *t* and *z*_*t*_ ∈ {1,..., K} such that there are *K* states, *x*_*t*_ ∈ ℝ^M^ is a matrix of *M* predictors in the GLM part of the model at time *t*, and **w**_*k*_ ∈ ℝ^M^ represents *M* GLM weights over predictors that are specific to state *k*. Transitions between the states unfold according to a transition matrix *A* ∈ ℝ^*K*×*K*^

$$p\left(z_{t+1}=k|z_{j},A\right)=A_{j,k}$$

where transition matrix is stationary. The joint probability of behaviors and HMM states is given by

$$p\left(y,z|{\left\{x\right\}}_{t=1}^{T},{\left\{w\right\}}_{k=1}^{K},A,\tau \right)=p\left(z_{1}\right)\prod_{t=2}^{T}p\left(z_{t+1}|z_{t}\right)p\left(y_{t},x_{t}\right)$$

where τ ∈ ℝ^*K*^ is the initial state distribution such that $\tau_{k}=p\left(z_{1}=k\right)$ . The likelihood function was fit to behavioral data via Markov chain Monte Carlo optimization implemented in STAN (*67*). For fMRI sessions, GLM-HMMs were fit to each individual animal’s behavior. For TUS sessions, GLM-HMMs were fit for each stimulation condition.

We probed the explanatory benefit of the GLM–HMM framework by fitting a series of models with *K* ∈ {1, 2, 3, 4, 5} states. This involved a five-fold cross-validation protocol in which models were iteratively fit to 80% of sessions and tested on the remaining 20% of sessions (*18*). This process was performed within each animal. The best fitting model was determined on the basis of the log-likelihood of held-out sessions (see fig. S3). In Fig. 2, we further report session-wise Akaike Information Criterion (AIC) scores—a well-established and easily interpretable metric of model fit that penalizes model complexity and is a demanding test for GLM-HMMs with *K ≥* 2 because additional states increase complexity exponentially.

We further validated the GLM-HMM framework using simulated data. To begin, we tested whether the fitting procedure accurately recovered the parameters used to generate simulated datasets by taking a fitted binomial GLM (i.e., *K* = 1) and two-state GLM-HMM for an example animal and generating 10 datasets comprising 16 sessions from each model (i.e., the typical size of the behavioral data recorded from each animal). We then implemented the fitting procedure on simulated datasets and tested (i) whether the correct number of HMM states was recovered (one state versus two states), and (ii) whether the correct parameters were identified in the two-state case (see fig. S8). We additionally examined simulated data for qualitative patterns that were reminiscent animal behavior (Fig. 3). After model selection, we decoded the maximum a posteriori sequence of HMM states in each session via the Viterbi algorithm (*67*)

$$z_{s}^{*}={\text{argmax}}_{z1:T}p\left(z_{s,1:T}|y_{s,1:T}\right)$$

We used simulated data from a two-state GLM-HMM to validate the Viterbi decoding algorithm (Fig. 4, A to C). Decoding the HMM states allowed us to quantify their impact on behavior, which we did using the following mixed-effects GLMs with subject identity as the grouping variable (GLM2.1)

$$\begin{array}{c} \text{logit}\left({\text{decision}}_{t}\right)=\beta_{o}+\beta_{1}{\text{reward-magnitude}}_{t}\\ +\beta_{2}{\text{reward-probability}}_{t}+\beta_{3}{\text{motivation-level}}_{t}\\ +\beta_{4}\;\text{reward-}{{\text{magnitude}}_{t}}^{*}{\text{reward-probability}}_{t}\\ +\beta_{5}{\text{trial-number}}_{t}+\mu_{0}+\mu_{1}{\text{motivation-level}}_{t}+\varepsilon \end{array}$$

where β_0–5_ are fixed-effects, μ_0–1_ are by-subject random effects for intercepts and motivation level, respectively, and ε is an error term. Motivation-level ∈ {0,1} is a binary variable reflecting the motivation state (low versus high) occupied on trial *t*. We identified motivation-state transitions by searching for cases where decoded motivation states were different across consecutive trials (i.e., motivation-level_*t*_ ≠ motivation-level_*t*–1_). We compared behavior before-versus-after motivation-state transitions with the following mixed-effects GLM (GLM2.2):

$$\begin{array}{c} \text{logit}\left({\text{decision}}_{t}\right)=\beta_{o}+\beta_{1}{\text{reward-magnitude}}_{t}\\ +\beta_{2}{\text{reward-probability}}_{t}\\ +\beta_{3}{\text{before-versus-after-transition}}_{t}\\ +\beta_{4}\;\text{reward-}{{\text{magnitude}}_{t}}^{*}{\text{reward-probability}}_{t}\\ +\beta_{5}{\text{trial-number}}_{t}\;\mu_{0}+\mu_{1}{\text{motivation-level}}_{t}+\varepsilon \end{array}$$

where β_0–5_ are fixed-effects, μ_0–1_ are by-subject random effects for intercepts and time from transition, respectively, and ε is an error term. Before-versus-after transition was a binary variable that grouped trials into two categories: (i) “before-transition” trials, defined as the five trials preceding a motivation-state transition, and (ii) “after-transition” trials, defined as the five trials in the immediate aftermath of a transition. We performed GLM2.2 separately for low-to-high and high-to-low motivation-state transitions. We then characterized the GLM-HMM’s convergent validity by comparing decoded states with variables that should change with motivation: (i) reaction-time, and (ii) pupil-size. To do this, we implemented the following mixed-effects GLMs (GLM2.3 and GLM2.4)

$$\begin{array}{c} \text{log}\left({\text{Reaction-time}}_{t}\right)=\beta_{o}+\beta_{1}\text{reward-magnitude}\\ +\beta_{2}\text{reward-probability}+\beta_{3}\text{HMM-state}\\ +\beta_{4}\text{trial-number}+\mu_{1}\text{HMM-state}+\varepsilon \end{array}$$

$$\begin{array}{c} {\text{Pupil-size}}_{t}=\beta_{o}+\beta_{1}\text{reward-magnitude}+\beta_{2}\text{reward-probability}\\ +\beta_{3}\text{HMM-state}+\beta_{4}\text{trial-number}+\mu_{1}\text{HMM-state}+\varepsilon \end{array}$$

where β_0–4_ are fixed effects and μ_1_ are random effects within each subject. We did not implement random intercepts (i.e., μ_0_) for either model because pupil size and reaction time were *z*-scored within subjects during preprocessing. This meant that the central tendency of within-subject distributions for reaction time and pupil size was the same value (i.e., 0), and it was not necessary to add terms capturing inter-subject differences.

We used changes in decoded HMM states to examine the temporal dynamics of motivation states. We were particularly interested in links between motivation states and aspects of an animal’s external environment, like the recent history of reward opportunities it had been presented with. We quantified the latter by calculating a moving average of the expectation value of reward opportunities received in the previous five trials.

$$\text{Ave}.{\text{EV}}_{t}=\text{mean}\left({\text{EV}}_{t–5}:{\text{EV}}_{–1}\right)$$

We used availability of rewards (Ave. EV) to predict motivation states via the following mixed-effects binomial GLM (GLM2.5)

$$\begin{array}{c} \text{logit}\left({\text{motivation-state}}_{t}\right)\in \left\{0,1\right\}=\beta_{o}\\ +\beta_{1}\text{EV}+\beta_{2}\text{Ave}.\text{EV}+\beta_{3}\text{trial-number}+\mu_{0}+\mu_{1}\text{Ave}.\text{EV}+\varepsilon \end{array}$$

where β_0–3_ are fixed effects and μ_0–1_ are random effects within each subject. We used the EV—the expected value of the current offer on a given trial—to control for the effects of the current offer on an animal’s motivation state. We used EV rather than the separate reward-magnitude and reward-probability dimensions of offers because Ave. EV and reward probability were meaningfully correlated (*r* = 0.49). This correlation was an inherent feature of the task because reward environments were engendered by manipulating the distribution of reward probabilities over time. This created autocorrelations in reward probabilities over time and, therefore, correlations between reward-probability and retrospective summary statistics of task events like the Ave. EV. Scaling reward probabilities by reward magnitude as in the expectation-value formula dampened this correlation (*r* = 0.23), meaning that EV and Ave. EV could be part of the same regression without collinearity.

### Acquisition, reconstruction, and preprocessing of MRI data

During collection of fMRI data, the monkeys were head-fixed in a sphinx posture within an MRI compatible chair by Rogue Research. MR images were acquired with a horizontal bore clinical 3T scanner with a 15-channel nonhuman primate–specific receive coil by RAPID Biomedical. Structural images were acquired during a previous experiment (*36*). Functional images were acquired via the CMMR multiband gradient-echo T2* echo planar imaging (EPI) sequence designed specifically to achieve high signal-to-noise in subcortical structures (*68*, *69*). This was characterized by 1.25-mm isotropic voxels with a repetition time (TR) of 1282 ms, echo time (TE) of 25.40 ms, multiband acceleration factor MB = 2, in-plane acceleration factor R = 2, and flip angle of 63°. The functional scans covered the whole brain (field of view, 120 mm). There were 42 slices at coronal orientation with foot-to-head phase encoding direction and 1.25-mm thickness.

Offline reconstruction of the raw functional data was performed following the dynamic off-resonance correction method developed by Shahdloo and colleagues (*70*). In summary, standard Nyquist ghost correction and dynamic zeroth-order B0 correction were applied first. Then, the EPI reference navigator data acquired at every time point were compared to navigator data from single-band references to estimate first-order dynamic off-resonance perturbations arising from the awake animal’s body movements. Last, the off-resonance estimates were used to correct the raw data before reconstruction.

Preprocessing of MR images was performed with a combination of FMRIB’s software library, Advanced Normalization Tools, the Human Connectome Project Workbench, and Magnetic Resonance Comparative Anatomy Toolbox (*71*). Although monkeys were head-fixed during MRI acquisition, incidental limb and body movements caused time-varying distortions in the B_0_ magnetic field and therefore nonlinear motion artefacts along the phase encoding direction. To account for this, a low-noise EPI volume was identified for each session and then implemented as a reference to which other volumes were nonlinearly registered slice by slice along the phase-encode direction. Aligned and distortion-corrected EPIs were then registered nonlinearly first to monkey-specific high-resolution images and then to a group template in CARET f99 macaque space. Further details of the group template construction are described elsewhere (*37*). Last, the functional images were temporally filtered (high-pass temporal filtering, 3-dB cutoff of 100 s) and spatially smoothed (Gaussian spatial smoothing, full-width half maximum of 2.5 mm).

Three measures were used to detect artefacts in the data: (i) For each slice in each volume the linear transform (in the *y* plane) from that slice to the corresponding slice in the mean reference image; (ii) the normalized correlation between that slice and the corresponding slice in the mean reference image; (iii) for each volume, the correlation between that volume (mean-filtered across *z* slices) and the mean reference image after correction. Volumes were removed when they exceeded 2.5 SDs above the median of each measure. The threshold was chosen to keep the number of censored volumes less than 10% of the total volumes. We also added 13 principal components analysis components describing, for each volume, the warping from that volume to the mean reference image when correcting motion artefacts (i.e., they capture signal variability associated with motion induced distortion artefacts), as parametric regressors of noninterest that were not convolved in our GLMs.

### fMRI data analysis

We focused our analysis of fMRI data on circumscribed a priori ROIs comprising DRN, VTA, SN, NB, LC, and Hb in the subcortex, and ACC, AI, and SMA in the cortex. Subcortical and SMA ROIs consisted in anatomical masks that were drawn on a group structural template in CARET F99 macaque monkey space and then warped to individual structural and functional spaces by nonlinear transformation. These masks were constructed separately by two different assessors based on the Rhesus Monkey Brain Atlas (*72*) and then evaluated on for convergence across assessors. ACC and AI ROIs were defined as 3-mm spheres centered on the peak of functionally relevant activation contrasts obtained in previous studies (*9*, *36*).

We extracted the filtered time series of BOLD signal from each ROI. The extracted signals were then averaged, normalized, and up-sampled by a factor of 15 (*9*, *36*, *37*). The upsampled data were then epoched to 6-s time windows spanning 1 s before to 5 s after the appearance of the reward-opportunity stimulus on each trial. We then examined the relationship between behavior and brain activity with ordinary least squares GLMs performed at each time point in each epoch.

Inferential statistics for time-course GLMs were performed using a leave-one-out cross-validation procedure designed to estimate the peak regression coefficient in each session without selection bias (*9*, *36*, *37*). For each session *s* (*N* = 59), we determined the time point *t* at which the largest absolute-value regression coefficient occurred in the remaining *N* – 1 (i.e., 58) sessions. We restricted our search for *t* to a 4-s window from 1 s after to 5 s after the decision-making time—that is, a 4-s window centered on the mean macaque HRF. We then calculated the regression coefficient in session *s* at time *t*. We repeated this iteratively for each session, which yielded a series of 59 regression coefficients. We performed significance testing on regression coefficients with one-sample two-tailed *t* tests. To control for multiple comparisons, we applied the Bonferroni-Holm correction for any analysis performed on more than one ROI. For each GLM, we repeated this for each regressor in each ROI. All time course analysis was conducted in MATLAB using custom analysis scripts.

We implemented the following series of GLMs (GLM3.1, GLM3.2, GLM3.3, and GLM3.4)

$$\begin{array}{c} {\text{BOLD}}_{\text{ROI}}=\beta_{o}+\beta_{1}\text{reward-magnitude}\\ +\beta_{2}\text{reward-probability}+\beta_{3}\text{environment-richness}\\ +\beta_{4}\text{environment-stochasticity}+\beta_{5}\text{behavioral-history}\\ +\beta_{6}{\text{environment-richness}}^{*}\text{behavioral-history}\\ +\beta_{7}{\text{environment-stochasticity}}^{*}\text{behavioral-history}\\ +\beta_{8}\text{pupil-size}+\beta_{9}\text{trial-number} \end{array}$$

where BOLD_ROI_ indicates a *t*-by-*s* matrix containing time-series data for a given ROI (where *t* is trial, and *s* is time sample). Pupil size and trial number were added as confound regressors to control for the effects of arousal and fatigue, respectively.

$$\begin{array}{c} {\text{BOLD}}_{\text{ROI}}=\beta_{o}+\beta_{1}\text{reward-magnitude}\\ +\beta_{2}\text{reward-probability}+\beta_{3}\text{environment-richness}\\ +\beta_{4}\text{environment-stochasticity}+\beta_{5}\text{pupil-size}+\beta_{6}\text{trial-number} \end{array}$$

GLM3.2 was performed separately for subsets of trials in which animals pursued and rejected the previous opportunity, respectively.

$${\text{BOLD}}_{\text{ROI}}=\beta_{o}+\beta_{1}\text{decision}+\beta_{2}\text{pupil-size}+\beta_{3}\text{trial-number}$$

where decision is the pursue-versus-reject decision made on a given trial.

$$\begin{array}{c} {\text{BOLD}}_{\text{ROI}}=\beta_{o}+\beta_{1}\text{motivation-transition-period}\\ +\beta_{2}\text{motivation-state-level}+\beta_{3}\text{pupil-size}\\ +\beta_{4}\text{trial-number} \end{array}$$

where transition-period is a dummy-coded variable covering symmetric 7-trial windows (*t* – 3:*t* + 3) around decoded motivation-state transitions and motivation-state-level is the decoded motivation-state (high versus low) for a given trial. GLM3.4 was subsequently performed with 7-trial transition-periods aligned to from (*t* – 6:*t* + 0) to (*t* – 0:*t* + 6) with respect to decoded transitions to compare the timing of transition-related signals (see Fig. 6). It was also performed separately for low-to-high and high-to-low transition events (see Fig. 6).

We tested whether DRN coded the value of recently available rewards (Ave. EV) with the following GLM (GLM3.5)

$$\begin{array}{c} {\text{BOLD}}_{\text{DRN}}=\beta_{o}+\beta_{1}\text{EV}+\beta_{2}\text{Ave}.\text{EV}\\ +\beta_{3}\text{pupil-size}+\beta_{4}\text{trial-number} \end{array}$$

GLM3.5 was performed on data from DRN alone, as it was designed to test a specific hypothesis arising from the combined neural and behavioral data. We used EV to control for the effects of the currently available offer instead of separate reward-magnitude and reward-probability regressors as in previous GLMs of BOLD signal. This is due to collinearity between Ave. EV and reward-probability regressors (see GLM2.5 for further explanation).

We probed changes in connectivity as a function of richness of the environment using the following PPI-GLM (GLM3.6)

$$\begin{array}{c} {\text{BOLD}}_{\text{ROI}}=\beta_{o}+\beta_{1}{\text{BOLD}}_{\text{seed}}+\beta_{2}\text{PPI}\\ +\beta_{3}\text{environment-richness}+\beta_{4}\text{reward-magnitude}\\ +\beta_{5}\text{reward-probability}+\beta_{6}\text{environment-stochasticity}\\ +\beta_{7}\text{pupil-size}+\beta_{8}\text{trial-number} \end{array}$$

where BOLD_seed_ is a *t*-by-*s* matrix containing time-series data for seed regions in PPI analysis, and PPI is the interaction between BOLD_seed_ and environment-richness regressors. GLM3.6 was performed separately for subsets of trials in which an animals pursued and rejected the previous opportunity, respectively. Analogously, we tested differences in functional connectivity with respect to (i) motivation-state level, and (ii) motivation-state transitions with the following PPI (GLM3.7)

$$\begin{array}{c} {\text{BOLD}}_{\text{ROI}}=\beta_{o}+\beta_{1}{\text{BOLD}}_{\text{seed}}\\ +\beta_{2}\text{PPI}+\beta_{3}\text{motivation-transition-period}\\ +\beta_{4}\text{motivation-state-level}+\beta_{5}\text{pupil-size}\\ +\beta_{6}\text{trial-number} \end{array}$$

where BOLD_seed_ is a *t*-by-*s* matrix containing time-series data for seed regions in PPI analysis. The analysis was performed twice—once where PPI was the interaction between BOLD_seed_ and motivation-transition-period regressors, and once where PPI was the interaction between BOLD_seed_ and motivation-state-level regressors. Last, we tested differences in functional connectivity with respect to pursue/reject decisions with the following PPI (GLM3.8)

$$\begin{array}{c} {\text{BOLD}}_{\text{ROI}}=\beta_{o}+\beta_{1}{\text{BOLD}}_{\text{seed}}+\beta_{2}\text{PPI}\\ +\beta_{3}\text{decision}+\beta_{4}\text{pupil-size}+\beta_{5}\text{trial-number} \end{array}$$

where BOLD_seed_ is a *t*-by-*s* matrix containing time-series data for seed regions in PPI analysis, and PPI is the interaction between BOLD_seed_ and decision regressors.

### Transcranial ultrasound stimulation

TUS was performed using a four-element annular array transducer (NeuroFUS CTX-500, 64-mm active diameter, Brainbox Ltd., Cardiff, UK) combined with a programmable amplifier (Sonic Concept Inc.’s Transducer Power Output System, TPO-105, Brainbox Ltd., Cardiff, UK). The transducer was paired with a transparent coupling cone filled with degassed water and sealed with a latex membrane. The water was degassed for 4 to 5 hours before each stimulation session and was replaced after each session. The resonance frequency of the ultrasonic wave was set to 500 kHz. The stimulation protocol was based on previously established protocols in macaques (*73*). We used the following protocol: duty cycle, 30%; pulse length, 30 ms; pulse repetition interval, 100 ms; and total stimulation duration, 30 s. The pressure field from the transducer was measured in a water tank with a 75-μm diameter polyvinylidene difluoride needle hydrophone (Precision Acoustics, Dorset UK), which had been calibrated at 500 kHz by the National Physical Laboratory (Teddington, UK). The free-field spatial-peak pulse-average intensity (Isppa) at 60-mm focal depth was 120 W/cm^2^, which was consistent with the output given by the transducer manufacturer.

At the beginning of each stimulation session, the animal’s skull was shaved and a conductive gel (SignaGel Electrode; Parker Laboratories Inc.) was applied to the skin. The water-filled coupling cone and the gel was used to ensure ultrasonic coupling between the transducer and the animal’s head. Next, the ultrasound transducer/coupling cone was placed on the skull and a Brainsight Neuronavigation System (Rogue Research, Montreal, CA) was used to position the transducer so that the focal spot would be centered on the targeted brain region. There were four stimulation conditions: (i) DRN (target of interest); (ii) VTA (subcortical active control condition); (iii) STS (cortical active control condition); (iv) sham (passive control condition). DRN and VTA targets were approximately 50 mm, and the STS 35 mm, from the surface of the transducer (the exact focal distance depended on the subject). All targets were sonicated bilaterally for 60 s in total, with 30 s of stimulation applied to a target from each hemisphere. Sonication of the midline targets (DRN and VTA) from one hemisphere was immediately followed by sonication of the same target from the contralateral hemisphere [cross-beam stimulation; (*36*)]. Sonication of the STS in one hemisphere was immediately followed by sonication of a homologous target in the contralateral hemisphere. Hemispheres were sonicated in a pseudo-random order. After stimulation, the monkeys were immediately moved to a testing room for behavioral data collection. The sham condition completely matched a typical stimulation session (setting, stimulation procedure, neuro-navigation, targeting, transducer preparation, and timing of its bilateral application to the shaved skin on the head of the animal) except that sonication was not triggered. During the sham session, the montage was pseudo-randomly positioned to target DRN, VTA, or STS. Each stimulation condition was repeated five times, on separate days, and the order of the stimulation sessions was pseudo-randomized for each animal. The stimulation was always performed at the same time of the day, and there was always a 24-hour gap between each session, regardless of it being a real or sham stimulation session.

### Acoustic modeling

We simulated the propagation of acoustic waves produced by the TUS protocol as described by Yaakub and colleagues (*74*). In brief, we used k-wave—a k-space pseudospectral solver (*75*)—and kArray tools to obtain estimates for the pressure amplitude, peak intensity, and spatial distribution of TUS at steady state. First, we simulated the acoustic wave propagation in water (free field) to characterize ultrasound beam for a target intensity of 120 W/cm^2^ at 50-mm focal depth (focal depth of VTA and DRN from transducer). Next, we performed the simulation for DRN and VTA targets in the skull. The skull was estimated from pseudo-CT images obtained from each monkey using a Black Bone MRI sequence (*76*). The skull was obtained by thresholding the pseudo-CT images at 1400 to 2100 Hounsfield Units (HU). A linear relationship between the pseudo-CT Hounsfield Units and the sound speed, as well as the density, and absorption coefficient was assumed as described elsewhere (*77*, *78*). The simulation grid size was set to the size of the T1-weighted MRI with a grid spacing of 0.5 mm, which results in approximately 6 points per wavelength in water and tissue and up to 12.4 points per wavelength in bone.

### Resting-state imaging data acquisition, preprocessing, and analysis

We further validated the TUS protocol by examining its effect on resting state coactivation patterns between VTA/DRN and key interconnected cortical and subcortical regions. Awake resting-state fMRI (rs-fMRI) data were acquired for all four monkeys (the same animals as in experiments 1 and 2) pre-versus-post DRN-TUS and VTA-TUS, respectively. Preprocessing and analysis of rs-fMRI data have been described elsewhere (*17*, *73*).

We characterized the effects of TUS on the coactivation of DRN/VTA and ROIs by comparing rs-fMRI data collected before DRN/VTA TUS with the rs-fMRI data collected immediately after DRN/VTA TUS. Prestimulation and poststimulation rs-fMRI were collected on the same day. The impact of DRN/VTA TUS on coactivation patterns was quantified with seed-based connectivity analyses, which involved calculating a series of pairwise linear correlations in BOLD activity between a seed region (DRN or VTA) and the remaining ROIs. The resulting prestimulation connectivity fingerprints for DRN-TUS and VTA-TUS were then contrasted with poststimulation connectivity fingerprints before DRN-TUS and VTA-TUS (see Fig. 7C).

### TUS data analysis

We characterized the behavioral effect of TUS by examining its two-way interactions with key predictors of decision-making in a series of binomial GLMs. In these GLMs, the effect for TUS condition was constructed to compare each individual active stimulation condition (STS, VTA, and DRN) to sham-TUS as a reference. On some occasions, we compared DRN-TUS to one of the other active stimulation conditions—for example, DRN-TUS versus STS-TUS. We did this by performing GLMs on subsets of data that included only the TUS conditions of interest. These analyses are specifically noted in the main text. We tested the following (GLM4.1, GLM4.2, GLM4.3, GLM4.4, and GLM4.5) to examine how TUS modulates the richness of the environment and behavioral history effects, respectively.

$$\begin{array}{c} \text{logit}({\text{decision}}_{t})=\beta_{o}+\beta_{1}\text{reward-magnitude}\\ +\beta_{2}\text{reward-probability}+\beta_{3}\text{environment-richness}\\ +\beta_{4}\text{environment-stochasticity}+\beta_{5}\;\text{TUS-condition}\\ +\beta_{6}\left({\text{reward-magnitude}}^{*}\text{reward-probability}\right)\\ +\beta_{7}\left({\text{environment-richness}}^{*}\text{TUS-condition}\right)\\ +\beta_{8}\left(\text{trial-number}\right)+\mu_{0}+\varepsilon \end{array}$$

where β_0–8_ are fixed effects, μ_0_ is the by-subject random intercept, and ε is an error term.

$$\begin{array}{c} \text{logit}\left({\text{decision}}_{t}\right)=\beta_{o}+\beta_{1}\text{reward-magnitude}\\ +\beta_{2}\text{reward-probability}+\beta_{3}\text{behavioral-history}\\ +\beta_{4}\text{TUS-condition}+\beta_{5}\left({\text{reward-magnitude}}^{*}\text{reward-probability}\right)\\ +\beta_{6}\left({\text{behavioral-history}}^{*}\text{TUS-condition}\right)+\beta_{7}\left(\text{trial-number}\right)+\mu_{0}+\varepsilon \end{array}$$

where β_0–7_ are fixed effects, μ_0_ is the by-subject random intercept, and ε is an error term. The amount of data in each TUS condition for each subject prevented us from fitting models with more complex random-effects structures for these models. We examined the influence of TUS on the frequency of transitions between motivation states with the following GLM

$$\begin{array}{c} \text{logit}\left({\text{state-transition}}_{t}\right)=\beta_{o}+\beta_{1}\text{reward-magnitude}\\ +\beta_{2}\text{reward-probability}+\beta_{3}\text{TUS-condition}\\ +\beta_{4}{\text{reward-magnitude}}^{*}\text{reward-probability}\\ +\beta_{5}\text{trial-number}+\mu_{0}+\varepsilon \end{array}$$

where, state-transition ∈ {0,1}, β_0–5_ are fixed-effects, μ_0_ is the by-subject random intercept, and ε is an error term. We performed a similar GLM to examine the influence of TUS on high-to-low transitions specifically

$$\begin{array}{c} \text{logit}\left({\text{state-decrease}}_{t}\right)=\beta_{o}+\beta_{1}\text{reward-magnitude}\\ +\beta_{2}\text{reward-probability}+\beta_{3}\text{TUS-condition}\\ +\beta_{4}{\text{reward-magnitude}}^{*}\text{reward-probability}\\ +\beta_{5}\text{trial-number}+\mu_{0}+\varepsilon \end{array}$$

where, state-decrease ∈ {0,1}, β_0–5_ are fixed-effects, μ_0_ is the by-subject random intercept, and ε is an error term. We analyzed TUS’s influence relationship between motivation states and the availability of rewards (Ave. EV) with the following GLM

$$\begin{array}{c} \text{logit}\left({\text{motivation-state}}_{t}\right)=\beta_{o}+\beta_{1}\text{EV}\\ +\beta_{2}\text{Ave}.\text{EV}+\beta_{3}\text{TUS-condition}\\ +\beta_{4}{\text{TUS-condition}}^{*}\text{Ave}.\text{EV}\\ +\beta_{5}\text{trial-number}+\mu_{0}+\varepsilon \end{array}$$

where, motivation-state ∈ {0,1} and the high-motivation state = 1 (i.e., positive coefficients correspond to increases in the likelihood of occupying the high-motivation state), β_0–5_ are fixed-effects, μ_0_ is the by-subject random intercept, and ε is an error term. We performed GLM4.5 separately on behavioral data mean-split according to Ave. EV to determine whether DRN-TUS principally affected the influence between low-value environments and low-motivation states.
