# Supplementary material for: Dorsal raphe nucleus controls motivation-state transitions in monkeys
Source: Sci Adv. 2025 Jun 27;11(26):eads1236. doi: 10.1126/sciadv.ads1236 (PMC12204389; doi:10.1126/sciadv.ads1236)
Supplement: Supplementary file 1 — Figs. S1 to S18 [file sciadv.ads1236_sm.pdf]

Science Advances
AAAS

Supplementary Materials for

Dorsal raphe nucleus controls motivation-state transitions in monkeys

Luke Priestley *et al.*

Corresponding author: Luke Priestley, luke.priestley@psy.ox.ac.uk

Sci. Adv. **11**, eads1236 (2025)
DOI: 10.1126/sciadv.ads1236

This PDF file includes:

Figs. S1 to S18

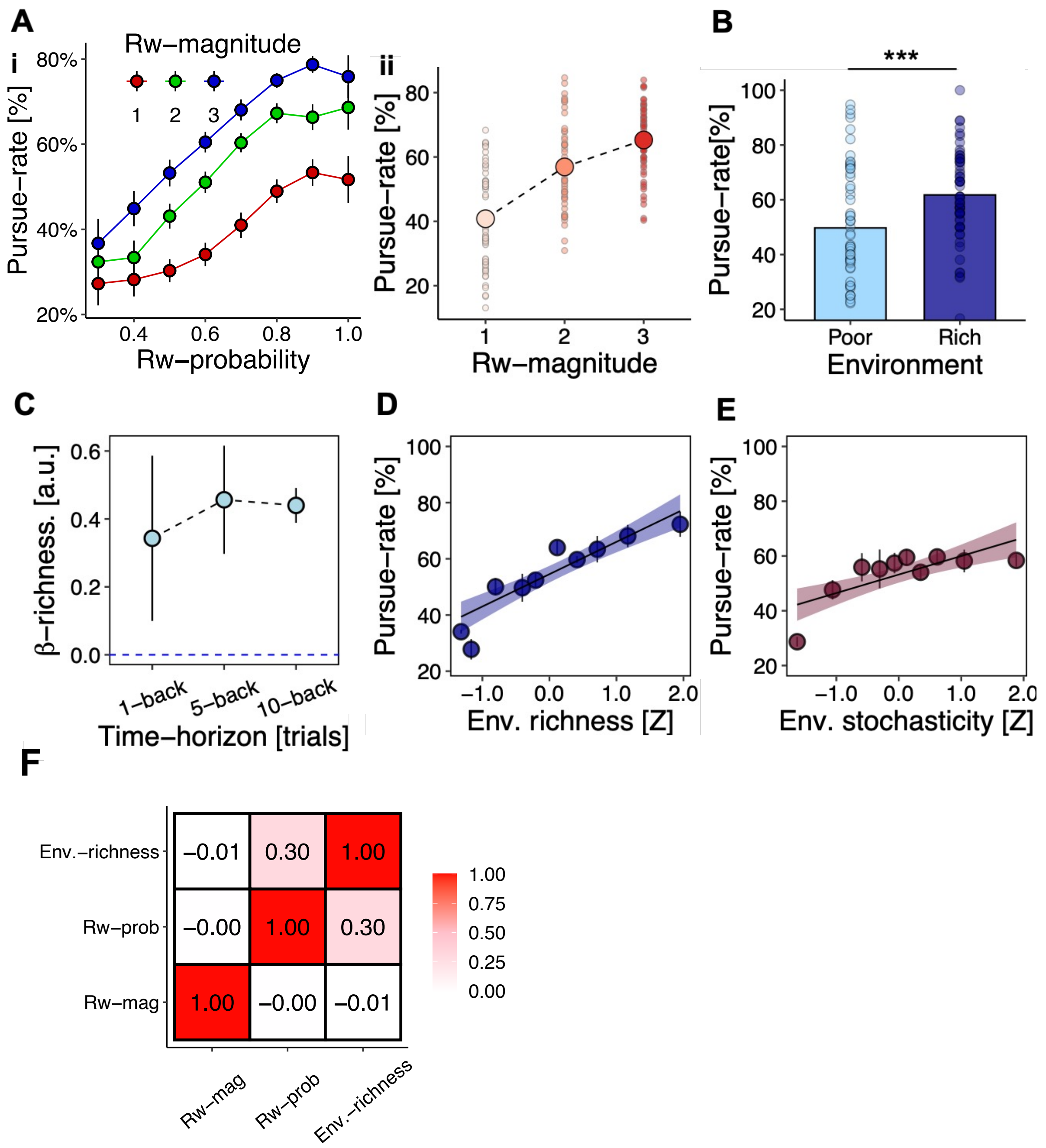

Figure S1. Additional analysis of behaviour. (**A**) Animals were more likely to pursue opportunities as the reward-probability **(i)** and reward-magnitude **(ii)** of the current offer increased (GLM1.2, see Methods; $\beta_{reward\text{-}probability}$=0.35, SE=0.23, *p*=<.001; $\beta_{reward\text{-}magnitude}$= 0.49, SE=0.02, *p*<.001). **(B)** The reward-probability distributions for rich-volatile and poor-volatile blocks partly overlapped (fig.1B), which produced a subset of trials with the same reward value in different contexts (poor vs rich). Animals were more likely to pursue offers that occurred in rich blocks relative to poor blocks, dovetailing with the analysis reported in the main text (fig.1E; $\beta_{rich\text{-}vs\text{-}poor}$=0.45, *SE*=0.19, *p*=.017). Dots indicate mean rates of responding in each session and animal. **(C)** In the main text, we report that animals are more likely to pursue reward opportunities as the richness of the environment increases, where richness of the environment is operationalised as the average reward accumulated in the previous five trials (see fig.1D for justification). Importantly, the same phenomenon occurs when the environment is operationalised using both shorter and longer time windows. We

tested two additional time-horizons: (i) where the richness of the environment was operationalised as the reward outcome on the preceding trial [β(environment-richness)=0.34, SE=0.12, p=.006; GLM3.4], and; (ii) where the richness of the environment was operationalised as the average reward outcome on the previous ten trials [β(environment-richness)=0.44, SE=0.03, p<.001; GLM3.4]. Y-axis shows effect-size for the richness of the environment in GLMs. X-axis indicates the time horizon of previous reward outcomes used to operationalise the richness of the environment. Points and whiskers indicate effect-size (i.e. regression-weight) ± 95%CI for each predictor. **(D) and (E)** Animals were more likely to pursue offers as the richness **(D)** and stochasticity **(E)** of the environment increased (see also fig.1E–F). Dots and whiskers indicate mean ± SEM of the pursue-rate in deciles of richness-of-environment and stochasticity-of-environment, respectively. **(F)** correlation between trial-by-trial values of reward magnitude, reward probability, and environment-richness predictors. The correlation matrix shows that these values are not collinear with one another.

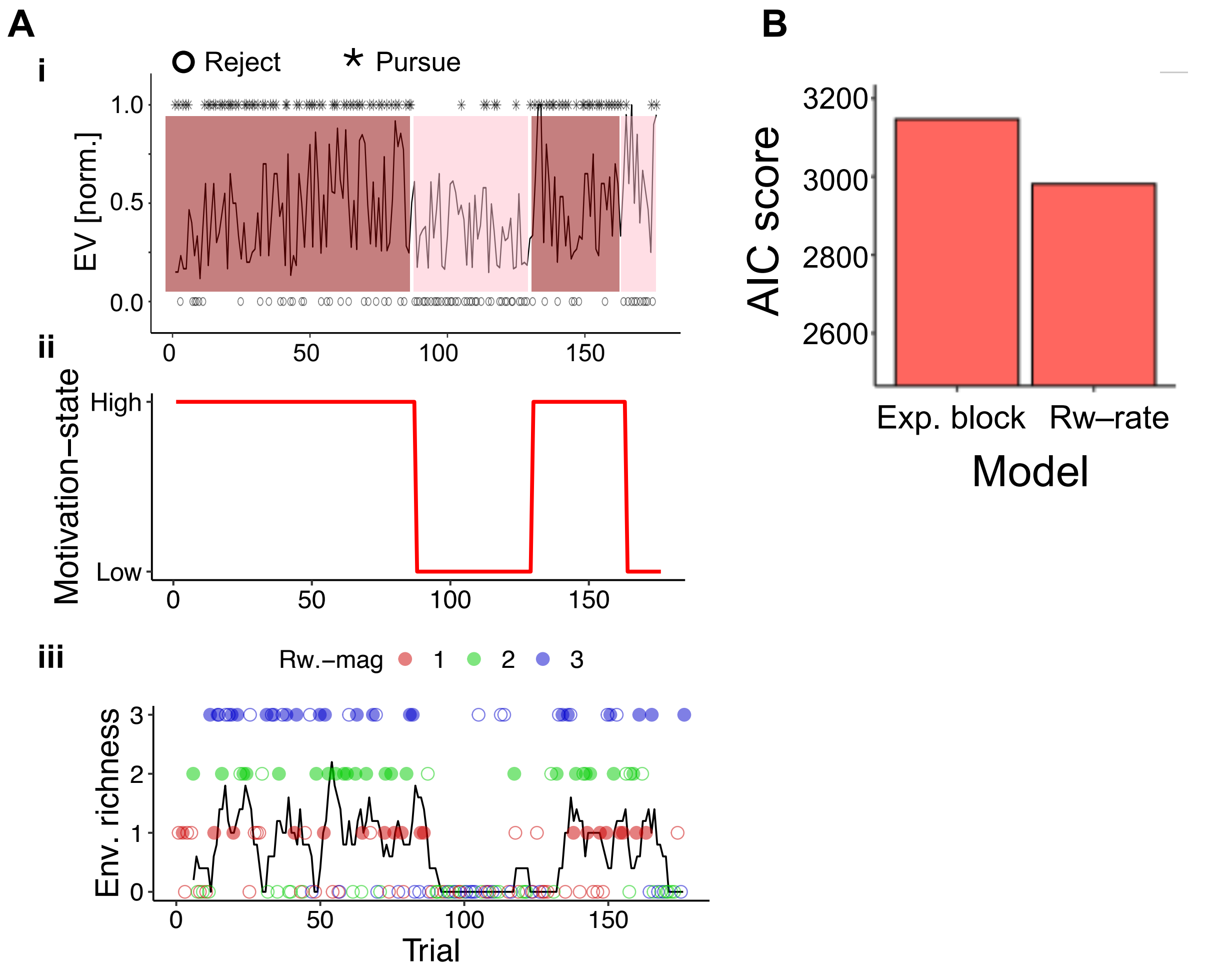

Figure S2. Comparing methods of operationalising the environment. (A) Fig.2A (shown here in panel **i**) shows that autocorrelations are visible in behavioural timeseries before any statistical analysis or pre-processing has been performed. For comparison, panel **ii** & **iii** show the decoded GLM-HMM motivation states (**ii**) and the reward-magnitude, response and environment-richness on each trial (**iii**) for the same session. In (**iii**), the black line indicates the environment-richness on each trial (i.e. average reward accumulated in the previous 5 trials). Coloured points indicate the magnitude of the reward opportunity on each trial. The colour fill indicates whether a reward was received on each trial – for example, a filled blue point indicates that the animal received a reward of magnitude three, whereas an unfilled point indicates that no reward was received. Unfilled data points at the bottom of the y-axis indicate opportunities that were not pursued. **(B)** Although the experimentally controlled structure of the task was characterised by discrete environments, we reasoned that a continuously varying reward-rate would more accurately capture an animal's experience of the task. In support of this view, we repeated the analysis reported in supplementary fig.S1B using two different models: (i) Model 1, where the environment was defined by the experimentally controlled rich-vs-poor blocks, and; (2) Model 2, where the environment is defined by the realised reward rate over the past five trials. Model 2 featuring the realised reward-rate, was clearly superior on AIC, suggesting that the realised reward-rate was a closer approximation of the way that animals understood the environment while performing the task. In the panel F, the x-axis indicates the two different models used in this analysis – Model 1, in which the

environment predictor reflects the experimentally controlled block-type, and Model 2, where it reflects the realised reward-rate. The y-axis indicates the AIC score of each model.

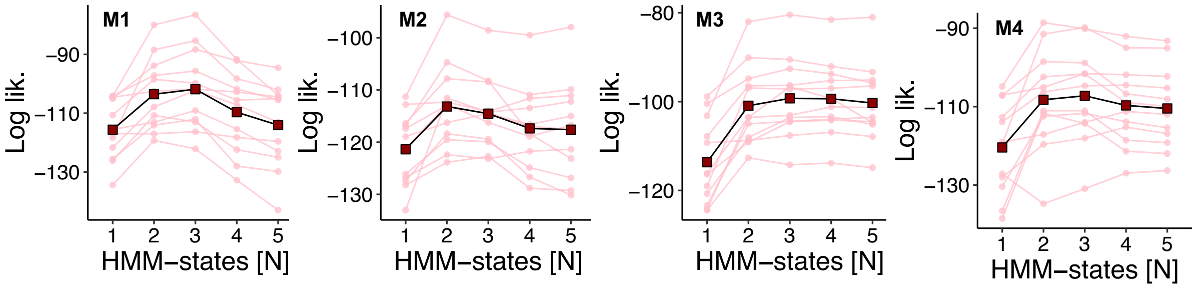

Figure S3. Cross-validation of the GLM-HMM. We tested GLM-HMMs using 5-fold cross-validation performed within each individual animal. This involved dividing the data into five folds and iteratively fitting the model to sessions in four folds (i.e. training folds) before testing performance on the held-out (i.e. test) fold. Our metric of model performance was log-likelihood, which we computed using the forward algorithm (see Methods). We constructed folds between-sessions instead of trials-within-sessions because between-session variance is greater than within-session variance (*18*), meaning that between-session cross-validation is a more demanding tests of performance. For each fold, we tested models with $s \in \{1, 2, 3, 4, 5\}$ HMM-states and calculated the log-likelihood of test sessions. All animals followed a similar pattern whereby models with two HMM-states performed better than Binomial GLMs (i.e. 1-state models), whereas additional states either failed to improve or impaired model performance. The relationship between the log-likelihood of the held-out sessions as a function of HMM-states is shown for all animals (foregrounded bold lines indicate mean log-likelihood of held-out sessions; background lines indicate log-likelihood of individual sessions).

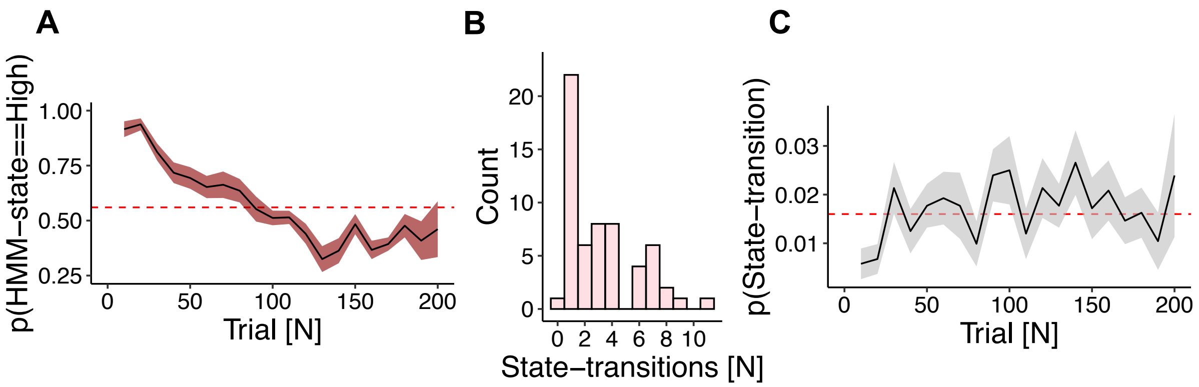

Figure S4. Further analysis of HMM-states. **(A)** The relative probability of motivation-states (y-axis) over time (x-axis). Animals are more likely to occupy high-motivation states early in a session, and approximately equally likely to occupy high-vs-low motivation states at the end of a session – motivation-state, thus, are not reducible to satiety, fatigue, or time-on-task. Black line and shaded areas indicate mean and SEM, respectively, of state-occupancy as a function of time; dashed red line indicates mean probability of motivation-state occupancy, regardless of time. **(B)** Histogram of the number of motivation-state transitions per session (x-axis). One session features no transitions. Data from all animals included. **(C)** The probability of motivation-state transition (y-axis) as a function of time (x-axis). Although transitions are more likely to occur in later trials, there is no consistent and specific temporal pattern in transition events indicating that they are not reducible to the passage of time (per (**B**)). Line and shaded area indicate mean and SEM, respectively, of state-transitions rates in quintile bins of trials. Dashed red line indicates overall mean probability of state-transition, regardless of time. Data from all animals included.

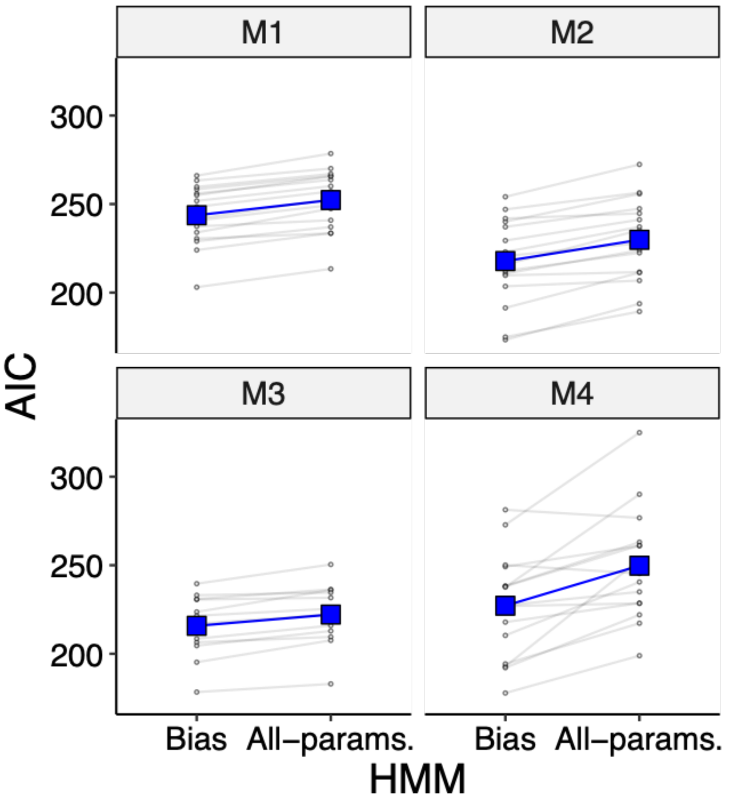

Figure S5. Comparison of alternative GLM-HMM models. In the main text, we focused on a GLM-HMM model where only the bias-parameter changed because we wanted to test a very specific hypothesis: that animals experienced changes in their baseline likelihood of pursuing rewards. In the GLM-HMM framework, this is best tested by allowing the bias/intercept parameter to change between HMM states. We reasoned that this was the simplest and most parsimonious way of quantifying whether animals displayed motivation-states. Even so, we have conducted additional analysis to compare two different GLM–HMMs; (1) a model where only the GLM bias parameter was allowed to change between HMM states, as reported in the manuscript, and; (2) a model where all GLM parameters were allowed to change between HMM states. In all four animals, the GLM–HMM where only the bias parameter was allowed to change performed the same as or better than the GLM–HMM in which all parameters were allowed to change. Importantly, the GLM–HMM where only the bias parameter was allowed to change had fewer total parameters than the GLM–HMM where all parameters were allowed to change. This means that even in cases where the performance of both models is equal, the GLM–HMM where only the bias parameter was allowed to change is a better explanation of the data because it is more parsimonious. X-axis shows model-type (‘bias’ indicates GLM–HMM where only the bias parameter changes, ‘all-params.’ indicates GLM–HMM where all parameters change), Y-axis shows Akaike Information Criterion (AIC), blue-points and blue line show mean session-wise AIC for each model-type, and grey points and lines show AIC for individual sessions for each model-type.

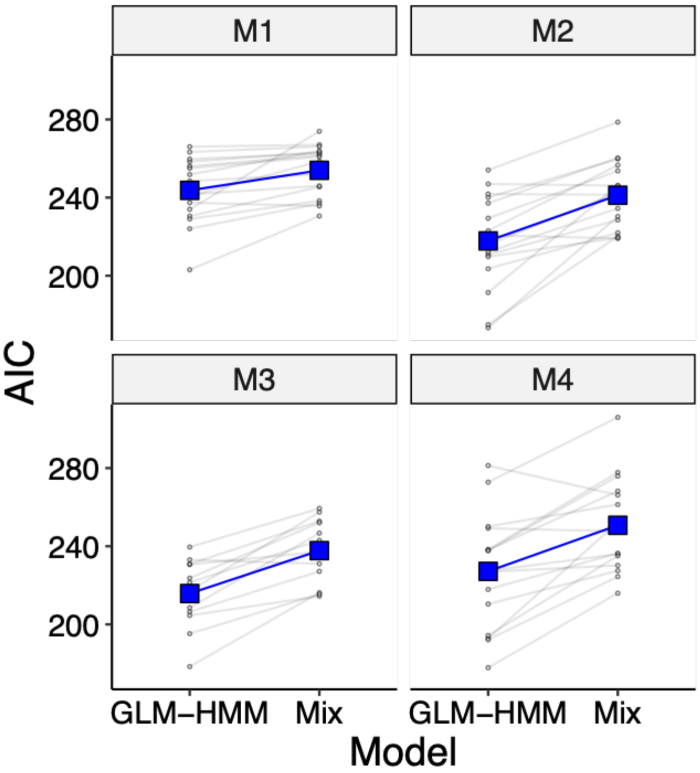

Figure S6. Comparison of 2-state GLM–HMM and mixture GLM. Animal behaviour likely arose from a mixture of distributions – one distribution where animals were predisposed to pursue rewards and another distribution where they were predisposed to reject rewards. This pattern suggests that a mixture GLM might successfully explain animal behaviour (*44*, *46*). However, because of the observed autocorrelation in behaviour, we believed that a model in which the mixture distributions evolve with Markovian dynamics, like a GLM–HMM, is more appropriate for our data than a mixture GLM with no Markov states. In support of this view, we compared the performance of the GLM-HMM to a mixture GLM, which indicated that the AIC of the data for each individual animal was higher for the GLM-HMM than the mixture GLM. X-axis shows model-type. Y-axis shows Akaike Information Criterion (AIC), blue-points and blue line show mean session-wise AIC for each model-type, and grey points and lines show AIC for individual sessions for each model-type.

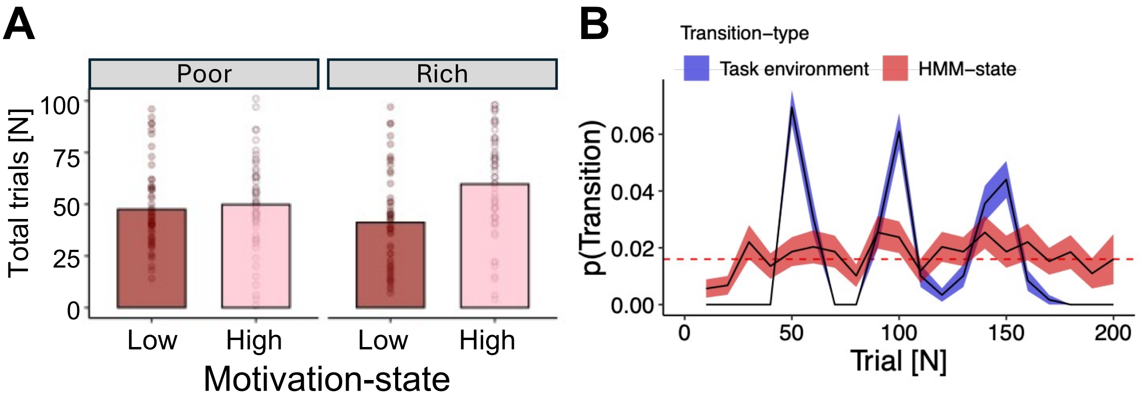

Figure S7. Relationship between HMM-states and experimentally programmed rich-vs-poor bocks. (A) X-axis indicates the different motivation-states identified with the GLM-HMM and the y-axis indicates the number of trials spent in that state in a session, where dots indicate data from individual sessions and bars indicate the mean number of trials spent in the motivation-state over all sessions. This suggests that although motivation-states are correlated with the experimentally programmed richness of the environment (e.g. animals are clearly more likely to occupy the high-motivation state in rich compared to poor blocks and more likely to occupy the low-motivation state in poor compared to rich blocks), there is variance in motivation-state occupancy that is not explained by the kind of experimentally programmed block an animal is in (e.g. animals spend a substantial number of trials in high-motivation sate in poor blocks and low-motivation state in rich blocks). **(B)** Visualising the relationship between transitions in motivation-states identified with the GLM–HMM and transitions between the X-axis indicates trial-number within each session and y-axis indicates the probability of transition on each trial (data is grouped into deciles along the x-axis for clarity). Different colours indicate the different kinds of transition that are recorded in the task: (i) in blue, transitions between experimentally programmed blocks, and; (ii) in red, transitions between motivation-states identified with the GLM-HMM. Lines and shadings show the mean ± SEM of the transition rate in each decile of trial-number along the x-axis.

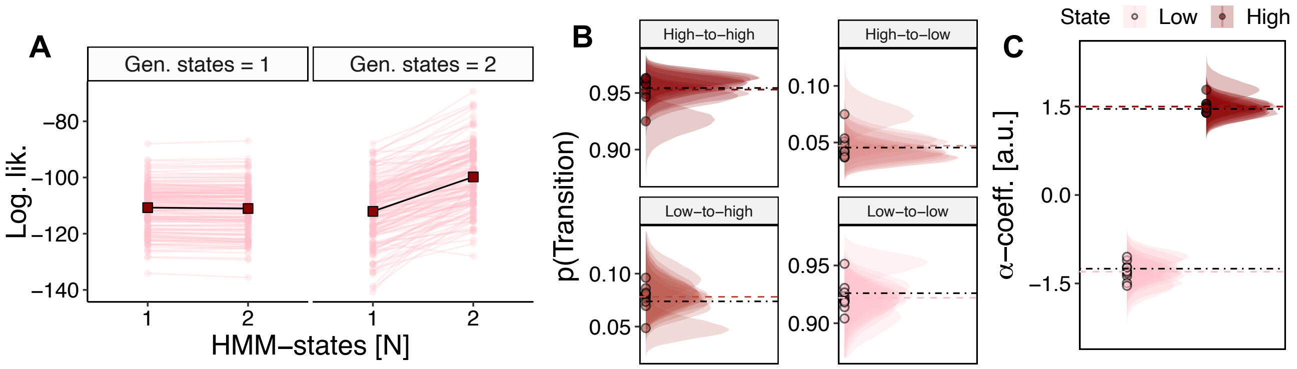

Figure S8. Parameter recovery for GLM-HMM. We assessed the performance of the GLM-HMM fitting procedure by simulating 10 datasets from fitted GLM-HMMs. Each dataset was approximately the same size as the total data obtained from each individual animal (~16 sessions, ~3000 trials). **(A)** We first tested whether the fitting procedure recovered the true number of HMM-states when the data was generated by a 1-state (i.e. Binomial GLM; left panel) and 2-state GLM-HMM (right panel) models. 2-state GLM-HMMs performed better under 2-state but not 1-state generative processes. All subsequent data pertains to simulations with 2-state GLM-HMM generative models. **(B)** The fitting procedure successfully recovered generative transition-matrix parameters. Graphs show posterior distributions over transition-matrix components in fitted 2-state GLM-HMMs. Black dashed lines indicate generative parameters and coloured lines indicate mean parameters over all fitted models. **(C)** The fitting procedure successfully recovered state-specific bias/intercept parameters. Graphs show posterior distributions over state-specific bias values. Dashed black lines indicate generative parameters and coloured lines indicate mean parameters over all fitted models.

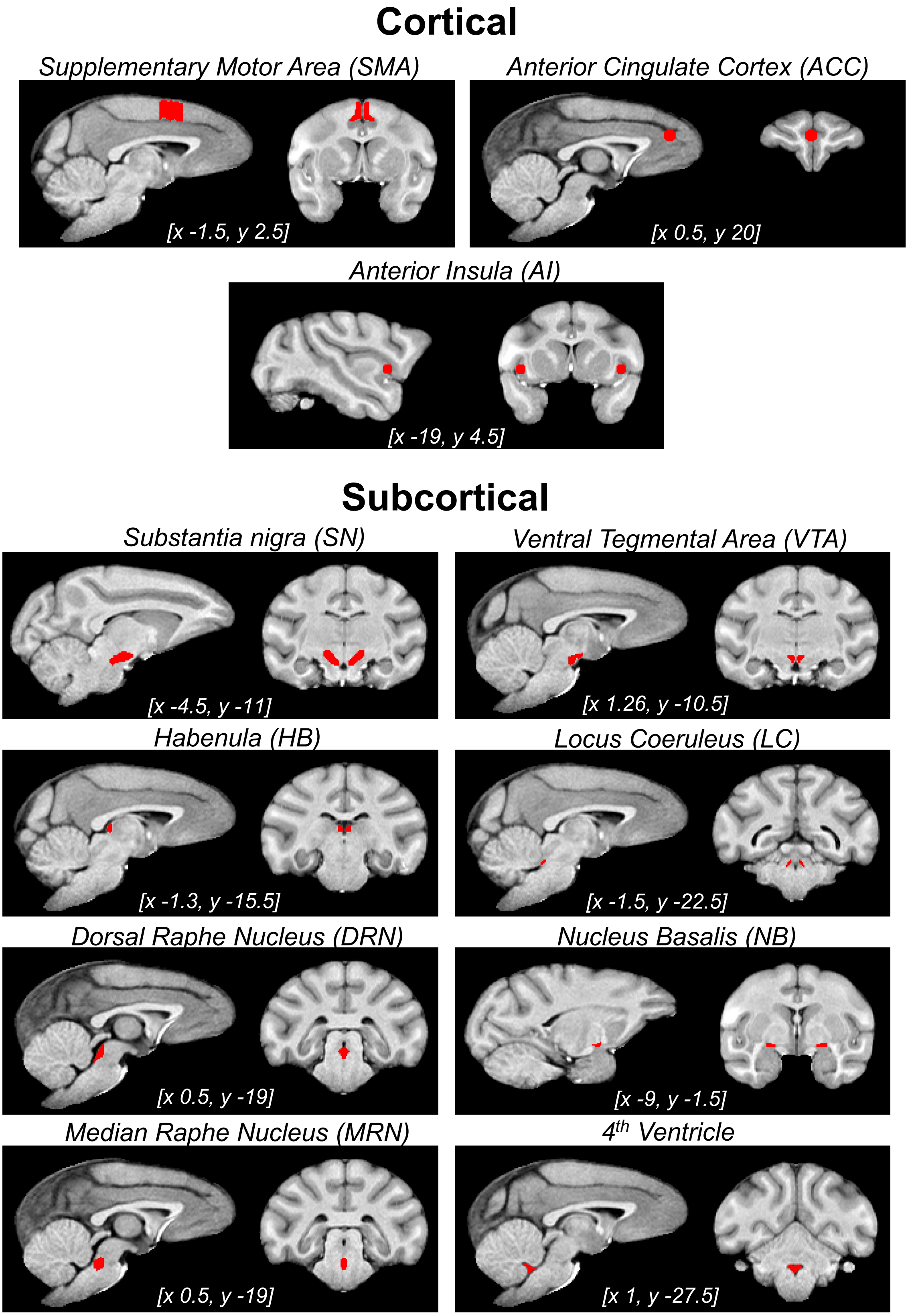

Figure S9. Cortical and subcortical regions of interest. An illustration of the ROIs implemented during fMRI analysis in CARET f99 macaque space. Subcortical and SMA ROIs consisted in anatomical masks that were drawn on a group structural template in CARET F99 macaque monkey space and then warped to individual

structural and functional spaces by nonlinear transformation. These masks were constructed separately by two-different assessors based on the Rhesus Monkey Brain Atlas *(72)* and then evaluated on for convergence across assessors. ACC and AI ROIs were defined as 3mm spheres centred on the peak of functionally relevant activation contrasts obtained in previous studies(*9*, *36*). The medial raphe nucleus (MRN) and fourth ventricle were only used as control ROIs to investigate the focality of the DRN effect.

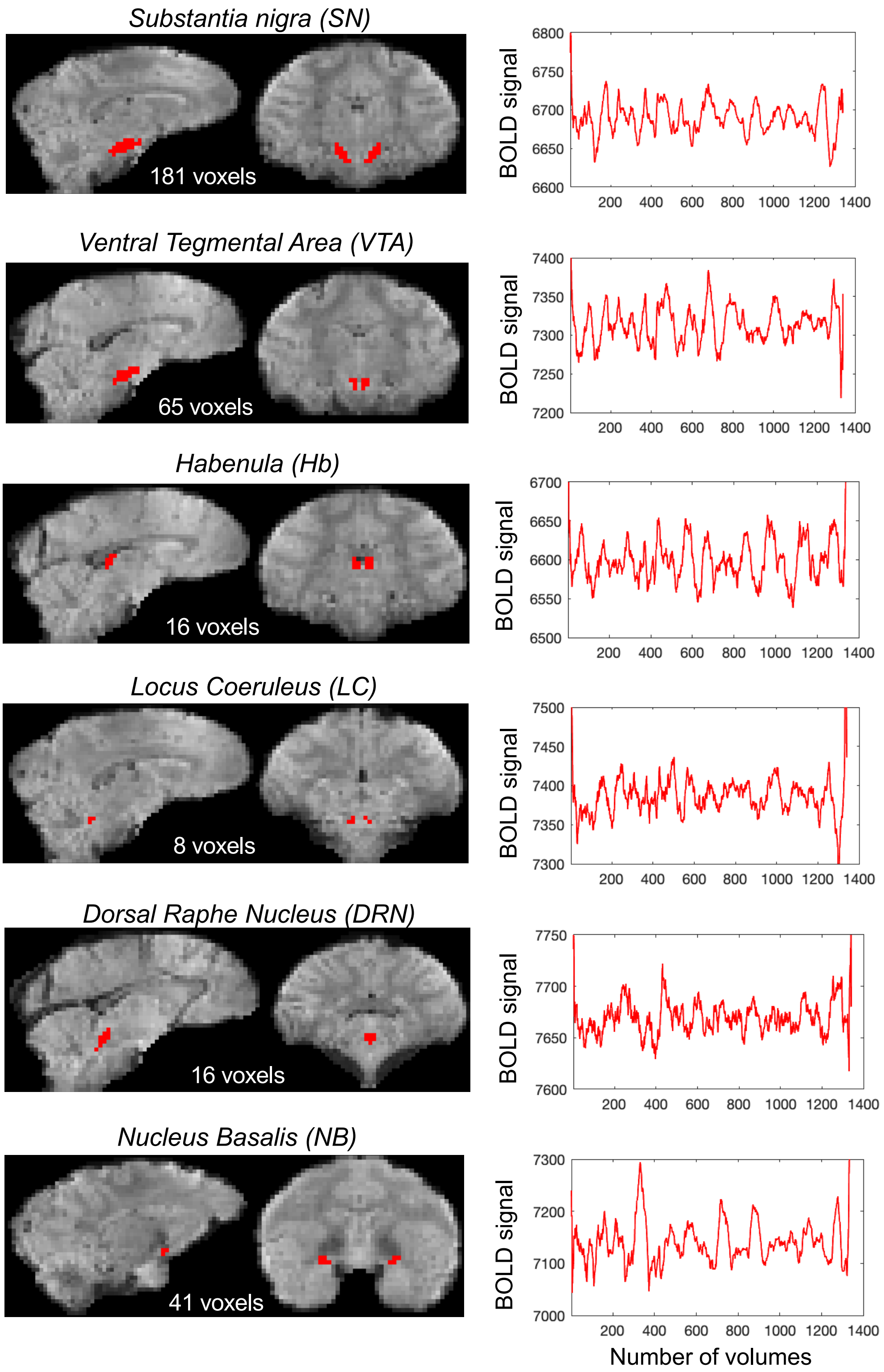

Figure S10. Anatomical masks from an example session in functional space. Anatomical masks were designed for each ROI in the standard space (see supplementary fig.S9) and were transformed from the standard space to each animal's functional space by applying a standard-to-structural-to-functional warp. The masks are overlaid on functional image from a representative session. The extracted BOLD signal from each mask is displayed next to its corresponding ROI. The lines show the

BOLD signal extracted and averaged from each voxel within the ROI, for the whole duration of the scanning session.

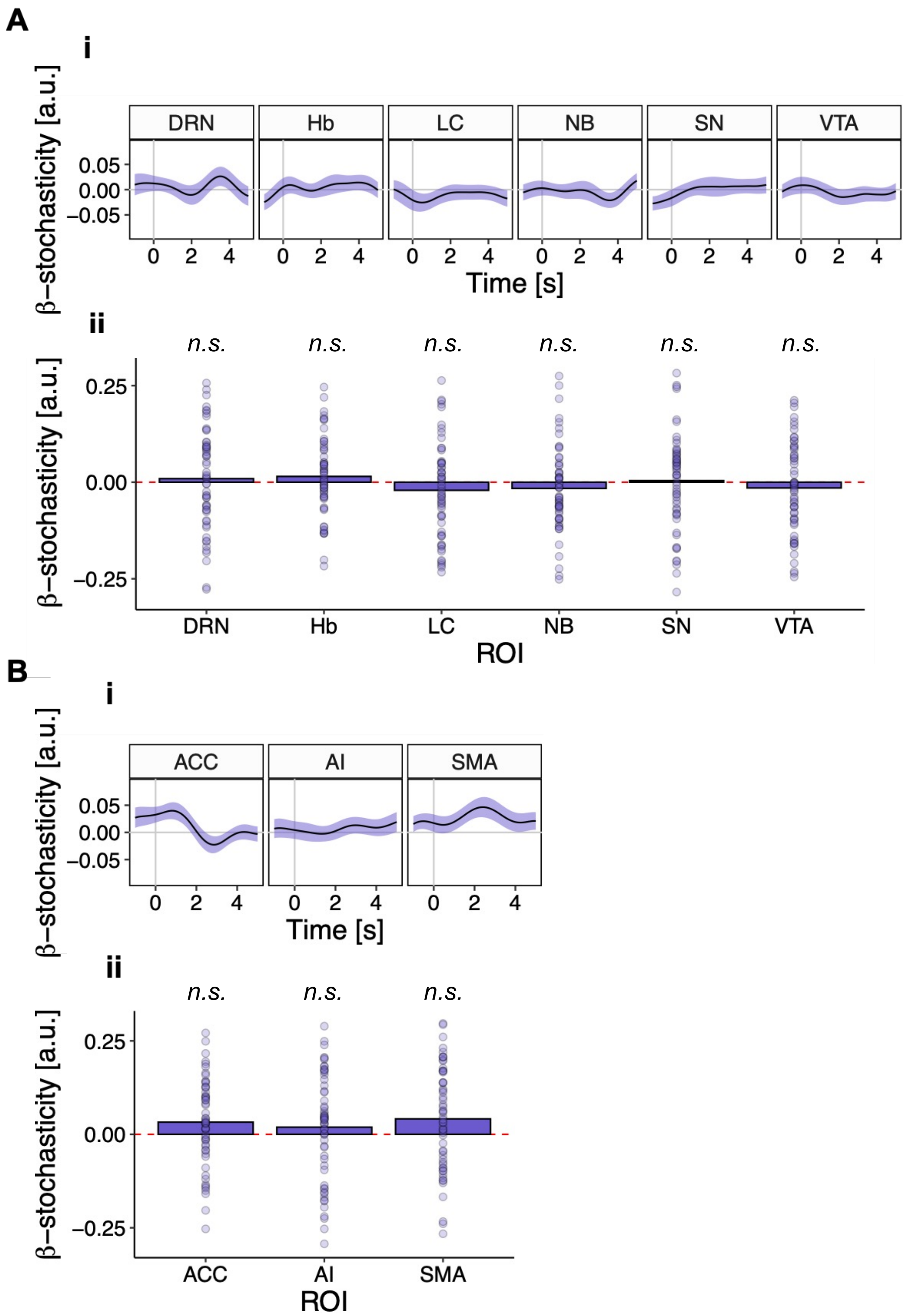

Figure S11. No evidence for environment-stochasticity representations in ROIs. The time-course and peak-regression-coefficients for the effect of reward-stochasticity on blood oxygen level dependent (BOLD) signal in subcortical **(Ai–ii)** and cortical **(B)** ROIs. Epochs are time-locked to decision-making. Reward-stochasticity had a modest influence on behaviour but there was no evidence that it was represented in the brain activity of ROIs (all *Ps*>0.05; after correction for multiple comparisons; GLM3.1).

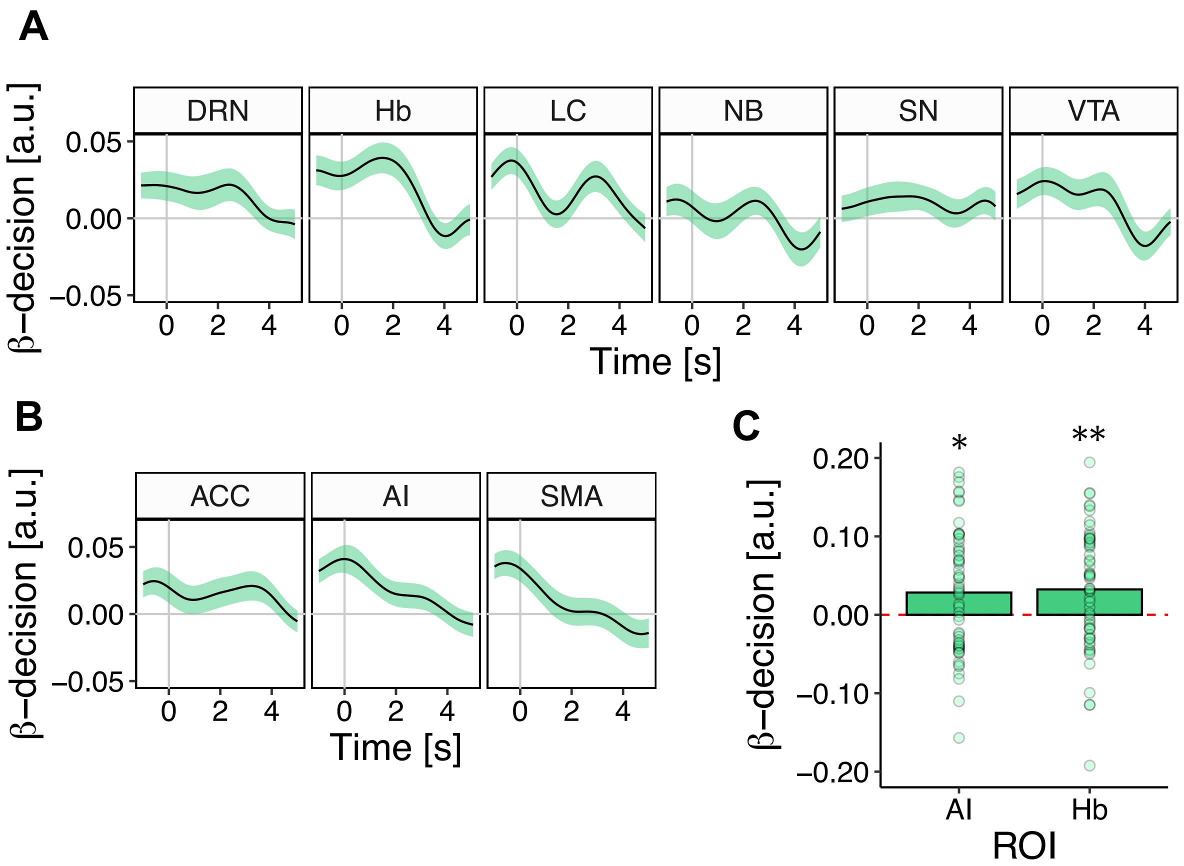

Figure S12. Habenula and Anterior Insula encode pursue/reject decisions. The time-course and peak-regression-coefficients of the relationship between pursuit decisions (pursue-vs-reject) and BOLD signal in subcortical **(A)** and cortical **(B)** ROIs. Epochs are time-locked to decision-making. **(C)** Pursuit decisions were represented in the BOLD activity of Hb ($t_{HB}(58) = 3.25$, $p = .012$) subcortically and AI ($t_{AI}(58) = 2.78$, $p = .022$) in the cortex. No other ROIs represented pursuit decisions.

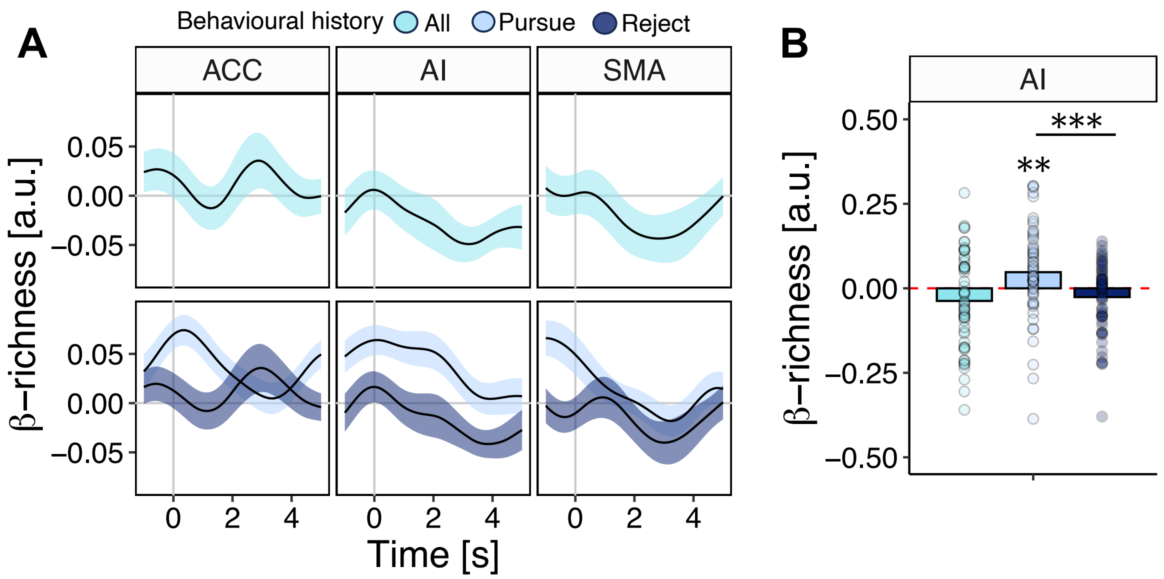

Figure S13. Anterior Insula represents the richness of the environment with the same pattern as subcortical ROIs. The time-course **(A)** and peak-regression-coefficients **(B)** of the relationship between richness of the environment and BOLD signal in cortical ROIs. All epochs are time-locked to decision-making. AI is the only cortical ROI to represent an animal's environment as a function of pursue/reject behaviour (GLM3.2; $t_{AI;\ rejected}(58)=-1.94$, $p=.057$; $t_{AI;\ pursued}(58)=3.02$, $p=.004$; $t_{AI;\ pursued\text{-}vs\text{-}rejected}(58)=3.73$, $p<.001$; Holm-Bonferroni correction not applied).

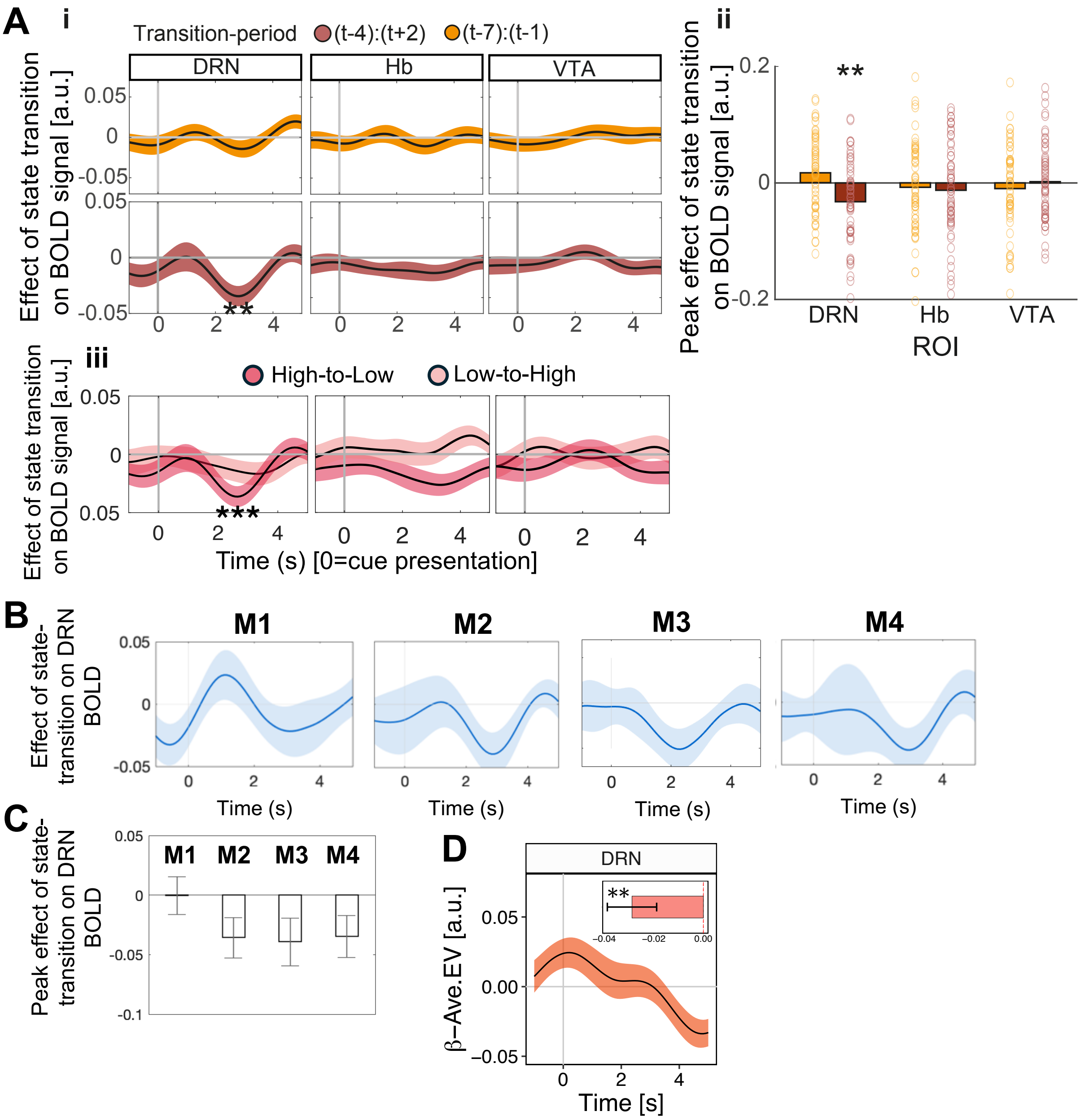

Figure S14. Further examination of motivation-state transitions in Dorsal Raphe Nucleus. (A) We initially tested the effect of motivation-state transitions on brain activity in a symmetric 7 trial 'transition-period' centred on the transition-trial (i.e. the transition trial +/- 3 trials). This effect, however, is robust as long as the transition-period includes the transition-trial itself. **(Ai)** shows that if we select a slightly earlier transition-period (t-4:t+2; red trace), we not only replicate our results, but the motivation-state transition effect becomes even stronger and clearer. However, if we shift the transition period even earlier so that it no longer includes the transition trial itself (t-7:t-1; orange trace), the effect disappears entirely. This underscores the reliability and robustness of the state-transition effect. **(Aii)** Distribution of peak effect sizes for motivation-state transition in a window that includes (red) and excludes (orange) the transition-trial itself, on brain activity. Dots indicate peak effect sizes for individual sessions. **(Aiii)** The timecourse of the effects shown in panel **Ai** for the t-4:t+2 transition period displayed separately for low-to-high (light pink) and high-to-low

(dark pink) motivation-state transitions. The effect is strongest in DRN for high-to-low state transitions. **(B&C)** The effect of motivation-state transitions on DRN BOLD is replicable in three out of four monkeys. **(D)** Our analysis of behaviour indicated that animals were more likely to occupy high motivation-states as the average expected-value of recently encountered rewards increased (fig. 4H). Given that BOLD activity in DRN represented transitions between motivation-states, we tested whether DRN also encoded the average expected-value of recent reward opportunities – the idea being that DRN might control changes in motivation-states specifically in relation to the distribution of available rewards. This was, indeed, the case – the average expected-value of the preceding five reward opportunities exerted a negative effect on BOLD activity in DRN ($t_{EV}(58)$ =-2.89, p=.005; GLM3.5). Inset panel shows distribution of peak regression coefficients.

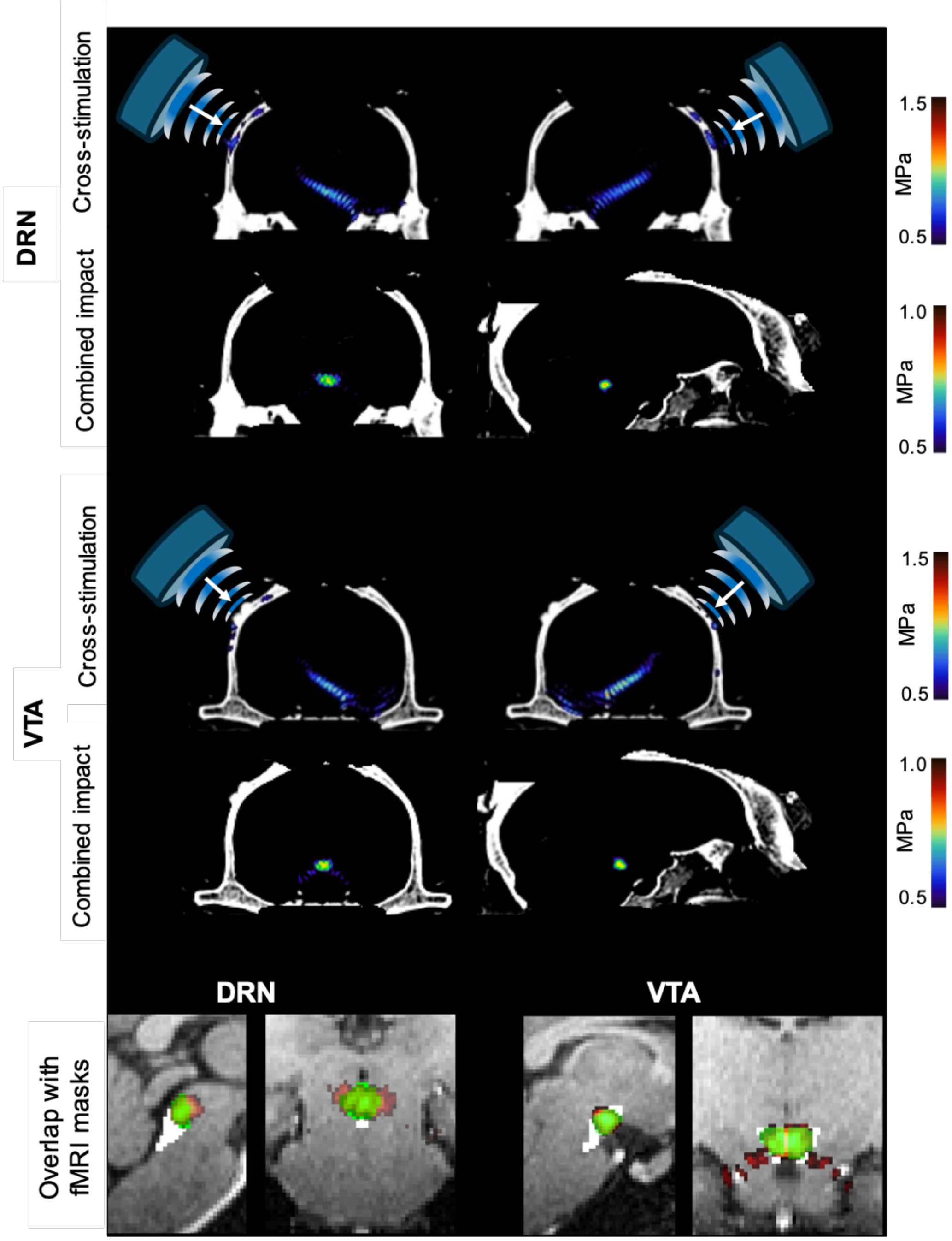

Figure S15. Simulation of the propagation of acoustic waves produced by the TUS protocol targeting DRN and VTA. Simulated focused ultrasound peak intensities and spatial distribution in the brain from successive left and right DRN / VTA stimulation. The skull was estimated from pseudo-CT images obtained from each monkey using a Black Bone MRI sequence *(76)*. The maximum spatial-peak pulse-averaged intensity (Isppa) at the acoustic focus point was 24.4 W/cm^2 (0.86 MPa) for the left DRN target, 21.5 W/cm^2 (0.80 MPa) for the right DRN target, 30.3 W/cm^2 (0.95 MPa) for the left VTA target and 19.9 W/cm^2 (0.77 MPa) for the right VTA target. The combined impact is calculated as the average stimulation intensity (I_{sppa}) map across the two consecutive stimulations delivered over the left and right hemispheres, for the DRN and VTA targets. The low impact probability level corresponds to 0.5 MPa (~8.3 W/cm^2), in correspondence with previous work *(36, 73)*. As the colour changes from blue to red, the probability of neuromodulation from bilateral DRN/VTA TUS increases.

The simulation procedure is described in the Methods and previous studies *(74)*. The simulated data shown here is from monkey M2. The cones show the position and trajectory of the ultrasound transducers. The white arrows show the direction of sound wave propagation. The bottom panel shows the DRN/VTA masks used in fMRI analysis in white, the ultrasound impact probability map in hot colours (thresholded at 0.5MPa, similar to upper panels and Fig.7B), and the overlapping voxels in green. The overlaid map shows a significant overlap between DRN and VTA masks used in fMRI analysis and the ultrasound impact probability map. Importantly, the expected peak impact of the stimulation lies within the fMRI masks for both DRN and VTA.

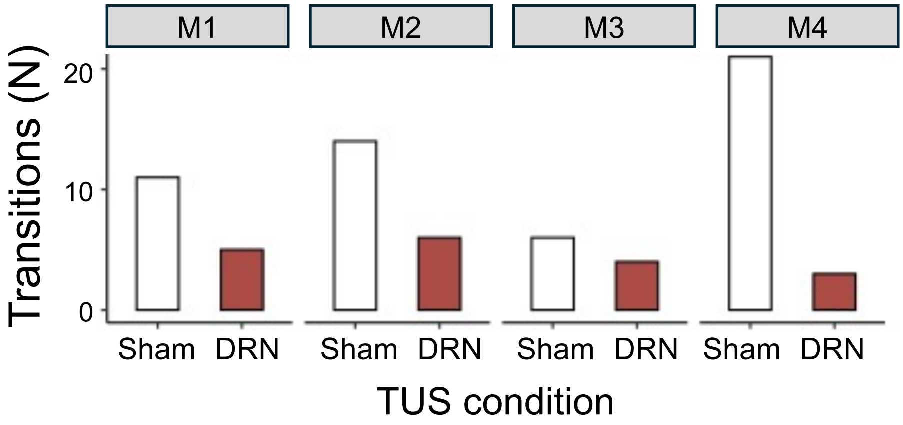

Figure S16. Animal-specific effect of DRN-TUS on motivation-state transitions We compared the number of motivation-state transitions exhibited by each separate animal in the DRN and sham TUS conditions. This comparison suggests that in all four animals, there is a reduction in the number of transitions exhibited in DRN-TUS sessions compared to sham sessions. In the figure, each panel shows motivation-state transition data for an individual animal. The x-axis indicates the TUS condition, and the y-axis indicates the number of motivation-state transitions observed (aggregated across all sessions within a given TUS condition).

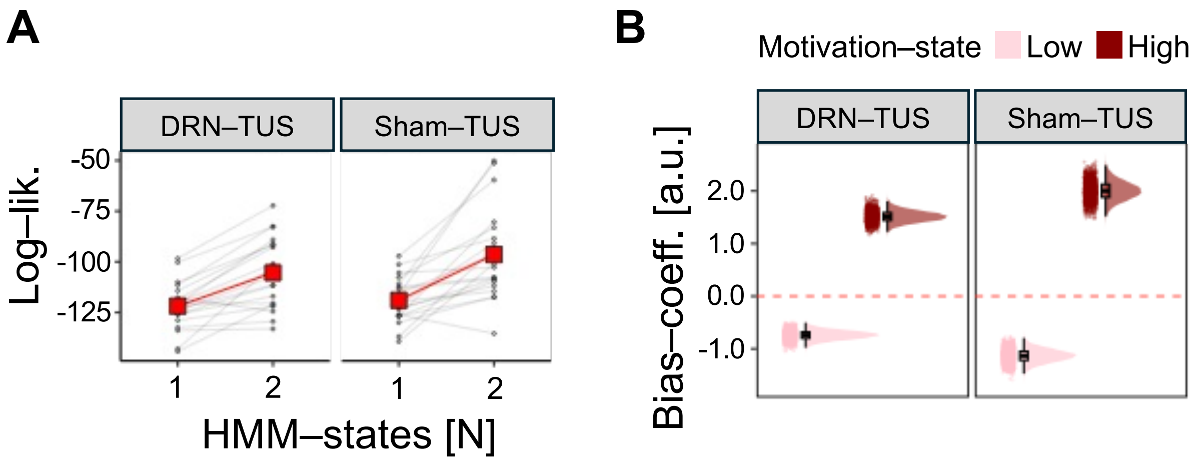

Figure S17. Evidence for two-motivation states after DRN-TUS. (A) A 2-state HMM-GLM model was clearly superior to a Binomial GLM in which there is one-state, in both DTN and sham-TUS conditions. **(B)** comparing the HMM-state-specific bias parameters in the GLM part of 2-state GLM-HMMs also showed that the HMM-state-specific bias parameters in the DRN-TUS condition were clearly divergent. However, in both **(A)** and **(B)**, HMM-states are slightly less distinguishable compared to the sham-TUS condition. In **(A)** the number of HMM-states in the model is shown on the x-axis, and the log-likelihood of the model is shown on the y-axis. Grey individual points show the log-likelihood of individual sessions, and comparison between the log-likelihood of the same session under different models is indicated by the grey lines. The mean session-level log-likelihood as a function of the number of HMM-states is shown in the red points and lines. In **(B)** the x-axis indicates different HMM-states from a 2-state GLM-HMM and the y-axis indicates values of HMM-state-specific bias parameters. The points, box charts and distributions indicate the posterior probability of HMM-state-specific bias parameter values.

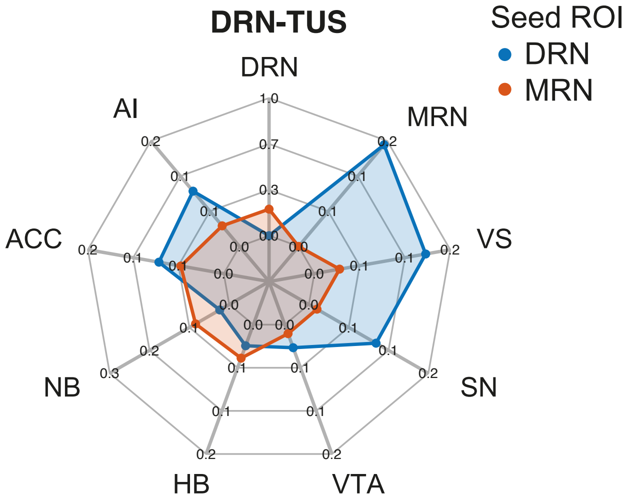

Figure S18. DRN-TUS effects do not arise from stimulation leakage. We have shown that the TUS effect is, as previously demonstrated (*36*) highly localised: DRN-TUS disrupted DRN's ordinary patterns of coactivation with key interconnected regions but left VTA connectivity unchanged, and the converse pattern occurred after VTA-TUS (fig.7C). However, the comparison cannot completely rule out potential leaking of stimulation to an adjacent structure closer to DRN but not DRN itself. We therefore performed a new analysis comparing DRN patterns of coactivation with MRN – a structure immediately adjacent to the DRN. While DRN-TUS disrupted DRN's ordinary patterns of coactivation with key interconnected regions it left MRN connectivity mainly unchanged. In panel D, the analysis follows the same format as in fig.7C. Radial axis shows absolute-value of the difference in connectivity between seed (DRN and MRN) and target ROIs pre-vs-post DRN disruption with TUS (DRN-TUS; see methods for details). Targets mainly focused on subcortical areas that are known to receive projections from MRN, including the basal forebrain (NB), habenula (Hb), ventral tegmental area (VTA), substantia nigra (SN), ventral striatum (VS), median raphe nucleus (MRN), dorsal raphe nucleus (DRN), anterior insular (AI), and anterior cingulate cortex (ACC).
